# Molecular characterisation of a novel pathogenic avipoxvirus from an Australian little crow (*Corvus bennetti*) directly from the clinical sample

**DOI:** 10.1038/s41598-022-19480-2

**Published:** 2022-09-05

**Authors:** Subir Sarker, Michelle Sutherland

**Affiliations:** 1 Department of Microbiology, Anatomy, Physiology and Pharmacology, School of Agriculture, Biomedicine and Environment, La Trobe University, Melbourne, VIC 3086 Australia; 2 The Unusual Pet Vets, 210 Karingal Drive, Frankston, VIC 3199 Australia

**Keywords:** Virology, Pox virus

## Abstract

Avipoxviruses are thought to be restricted to avian hosts and considered significant pathogens that may impact the conservation of many birds. However, reports of avipoxvirus-like viruses from reptiles suggest that cross-species transmission, within birds and other species, may be possible. The vast majority of avipoxviruses in wild birds remain uncharacterised and their genetic variability is unclear. Here, cutaneous pox lesions were used to recover a novel full-length crowpox virus genome from an Australian little crow (*Corvus bennetti*), followed by the detection of immature and intracellular mature virions using electron microscopy. The CRPV genome was 328,768 bp in length and contained 403 predicted open-reading frames. While 356 of the ORFs of CRPV genome had the greatest similarity with other avipoxviruses gene products, a further 47 ORFs were novel. Subsequent phylogenetic analyses showed that the CRPV was most closely related to other avipoxviruses isolated from passerine and marine bird species and demonstrated the highest sequence similarity with an albatrosspox virus (84.4%). Considering the sequence similarity observed between CRPV and other avipoxviruses and phylogenetic position, this study concluded that the CRPV to be a distinct available candidate of avipoxviruses.

## Introduction

Avipoxviruses are large, double-stranded DNA (dsDNA) viruses belonging to the genus *Avipoxvirus* (family Poxviridae, subfamily Chordopoxvirinae) that may cause proliferative, diphtheritic or systemic lesions in birds^1,2^. Avipoxviruses represent a diverse virus group, that may infect most avian species. Evidence of poxvirus infection has been found in at least 329 avian species and 20 orders of wild and domestic bird species^3,4^, with many more avian hosts considered susceptible. In general, avipoxviruses appear to have been present in bird populations for continuous periods, leading to low levels of infection and relatively mild disease. However, where poxviruses have been introduced to naïve bird populations, they have the potential to cause explosive outbreaks of severe disease with high morbidity and mortality, as occurred in Hawaii, the Galapagos and the Canary Islands^5,6^.

According to the International Committee on Taxonomy of Viruses (ICTV)^7^ there are currently 12 species approved under the genus *Avipoxvirus*: *Canarypox virus, Flamingopox virus, Fowlpox virus, Juncopox virus, Mynahpox virus, Penguinpox virus, Pigeonpox virus, Psittacinepox virus, Quailpox virus, Sparrowpox virus, Starlingpox virus* and *Turkeypox virus*. A further two viruses, crowpox virus and peacockpox virus*,* are putative members of the genus *Avipoxvirus*, but have not yet been approved as species by the ICTV. There are currently a limited number of complete avipoxvirus genome sequences from ICTV-recognised species available in GenBank. These include a canarypox virus (CNPV)^8^, a South African strain of pigeonpox virus (FeP2)^9^, a penguinpox virus (PEPV)^9^, a Hungarian strain of turkeypox virus (TKPV)^10^, an American strain of fowlpox virus (FWPV)^11^, a European strain of fowlpox virus (FP9)^12^, and an additional eight complete genome sequences of FWPV that have been published since 2018^13–15^. There are also six further complete avipoxvirus genomes: two shearwaterpox viruses (SWPV1 and SWPV2)^16^, two magpiepox viruses (MPPV and MPPV2)^17,18^, a mudlarkpox virus (MLPV)^19^, penguinpox virus 2 (PEPV2)^20^, two albatrosspox virus (ALPV and ALPV2)^21,22^, and a poxvirus in house finches (*Haemorhous mexicanus*)^23^ available in GenBank that are not yet ICTV recognised species.

Avipoxvirus infection in the family Corvidae has been identified in an adult American crow (*Corvus brachyrhynchos*) in the United States of America^24,25^. Several recent studies have also characterised poxvirus infections in the cutaneous lesions of Australian passerine bird species including the Australian magpie (*Gymnorhina tibicen*)^17,18^, and the mudlark (*Grallina cyanoleuca*)^19^. However, there are no sequence data of crowpox virus from the Australian little crow (*Corvus bennetti*), and consequently, its genetic and phylogenetic relationships with other avipoxviruses are not well known. The aim of this study was to identify and characterise the genome sequence of crowpox virus (CRPV) from an Australian little crow sourced from Victoria in 2021.

## Results

### Genome of crowpox virus (CRPV)

We determined the complete genome sequence of CRPV as a linear double-stranded DNA molecule of 328,768 bp in length (GenBank accession no. ON408417). The CRPV genome contained a large central coding region surrounded by two matching inverted terminal repeat (ITR) regions, constituting 4052 bp each (coordinates 1-4052 sense and 324,717–328,768 antisense orientation) like other characterised avipoxviruses^11,16,17,19,26^. Each of the inverted repeats constituted arrays of direct repeats, and six tandem repeats were detected within each inverted terminal repeat region. These consisted of a 103 bp, 85 bp, 60 bp, 9 bp and two 42 bp repeat unit and sharing approximately 97–100% nucleotide identity. The CRPV genome showed the highest nucleotide identity (84.4%) with the pathogenic avipoxvirus ALPV, isolated from an endangered northern royal albatross *(Diomedea sanfordi*) in 1997 (GenBank accession no. MW365933)^22^ (Table 1), followed by PEPV2 (84.3%), SWPV2 (83.4), CNPV (83.3%), and MPPV2 (82.6%). The A + T content of the CRPV genome was 71.3%, which was comparable to other sequenced avipoxviruses (Table 1).

**Table 1:** Comparative analysis of representative avipoxviruses and CRPV based on complete genome nucleotide sequences.

Avipoxviruses (abbreviation)	GenBank accession number	Genome identity (%)	Genome length (kbp)	A + T content (%)	Number of ORFs	References
Crowpox virus (CRPV)	ON408417		329	71.3	403	This study
Albatrosspox virus 2 (ALPV2)	OK348853	49.3	286	69.1	359	^21^
Albatrosspox virus (ALPV)	MW365933	84.4	352	71.2	336	^22^
Canarypox virus (CNPV)	AY318871	83.3	360	69.6	328	^8^
Fowlpox virus (FWPV)	AF198100	49.5	289	69.1	260	^11^
Flamingopox virus (FGPV)	MF678796	48.5	293	70.5	285	^3^
Finch poxvirus (FIPV)	OM869483	75.5	354	69.9	334	^23^
Magpiepox virus (MPPV)	MK903864	79.8	293	70.4	301	^17^
Magpiepox virus 2 (MPPV2)	MW485973	82.6	298	70.5	419	^18^
Mudlarkpox virus (MLPV)	MT978051	80.5	343	70.2	352	^19^
Penguinpox virus (PEPV)	KJ859677	50.7	307	70.5	285	^9^
Penguinpox virus 2 (PEPV2)	MW296038	84.3	350	69.9	327	^20^
Pigeonpox virus (FeP2)	KJ801920	49.9	282	70.5	271	^9^
Shearwaterpox virus 1 (SWPV1)	KX857216	62.0	327	72.4	310	^16^
Shearwaterpox virus 2 (SWPV2)	KX857215	83.4	351	69.8	312	^16^
Turkeypox virus (TKPV)	KP728110	35.3	189	70.2	171	^10^

### Genome annotation and comparative analyses of CRPV

**Figure 1:**
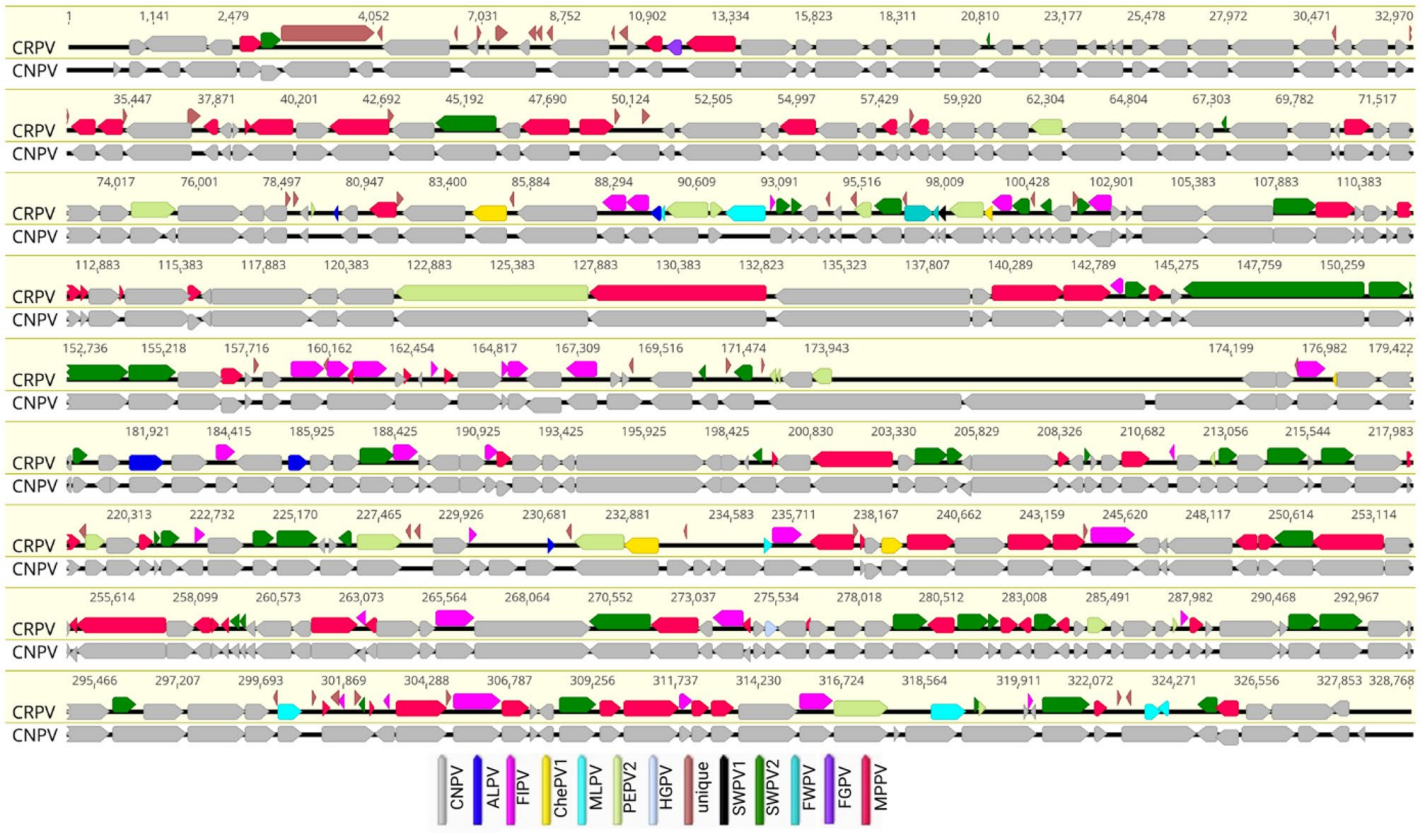
Genomic illustration of CRPV in comparison with CNPV was visualised using Geneious Prime^®^ (version 2022.1.1). The arrows depict the direction of transcription of genes and open reading frames (ORFs). Each ORF of CRPV genome is colour coded based on homology to other avipoxviruses, as indicated by the key in the legend.

The CRPV genome predicted to enclose 403 open reading frames (ORFs) encoding proteins ranging from 30 to 1945 amino acids in length, that were numbered from left to right (Fig. 1 and Supplementary Table S1). Among them, five predicted ORFs were found within the ITRs and were thus present as diploid copies. Comparative analysis of the predicted ORF sequences showed that 356 had the greatest similarity with other ChPV gene products (E value ≤ 10^−5^) (Fig. 1 and Supplementary Table S1). Among these predicted genes, 166 genes showed the highest similarity to a CNPV^8^, followed by 69 genes to MPPV^17^, 48 genes to SWPV2^16^, 32 genes to a recently sequenced FIPV^23^ and 19 genes to PEPV2^20^. A further seven (ORF-114, -117, -284, -363, -387, -397 and -398) showed highest similarity to MLPV, five (ORF-103, -113, -215, -219 and -279) to ALPV, five (ORF-108, -132, -210, -282 and -290) to ChePV1, two (ORF-128 and -129) to FWPV, one (ORF-329) to Hawaiian goose poxvirus (HGPV196), one (ORF-130) to SWPV1, and one (ORF-023) to FGPV (Fig. 1 and Supplementary Table S1).

Remarkably, the CRPV genome contained 47 predicted protein-coding genes that were unique based on the NR protein database using BLASTX and BLASTP^27^. These unique ORFs encoded proteins of 30 to 100 amino acids in length (Fig. 1 and Supplementary Table S1). Among them, 18 unique CRPV protein-coding ORFs (ORF-006, -007, -016, -017, -050, -060, -076, -099, -100, -124, -127, -138, -196, -276, -280, -283, -295, -366) were predicted to contain a single transmembrane helix, and software packages predicted that ORF-052 contained three transmembrane helices (Supplementary Table S1). We did not find any significant homology with known proteins for the unique ORFs encoded in the CRPV genome when using Phyre2, HHpred or SWISS-MODEL, which may result from the lack of closely related structures in these databases.

**Figure 2:**
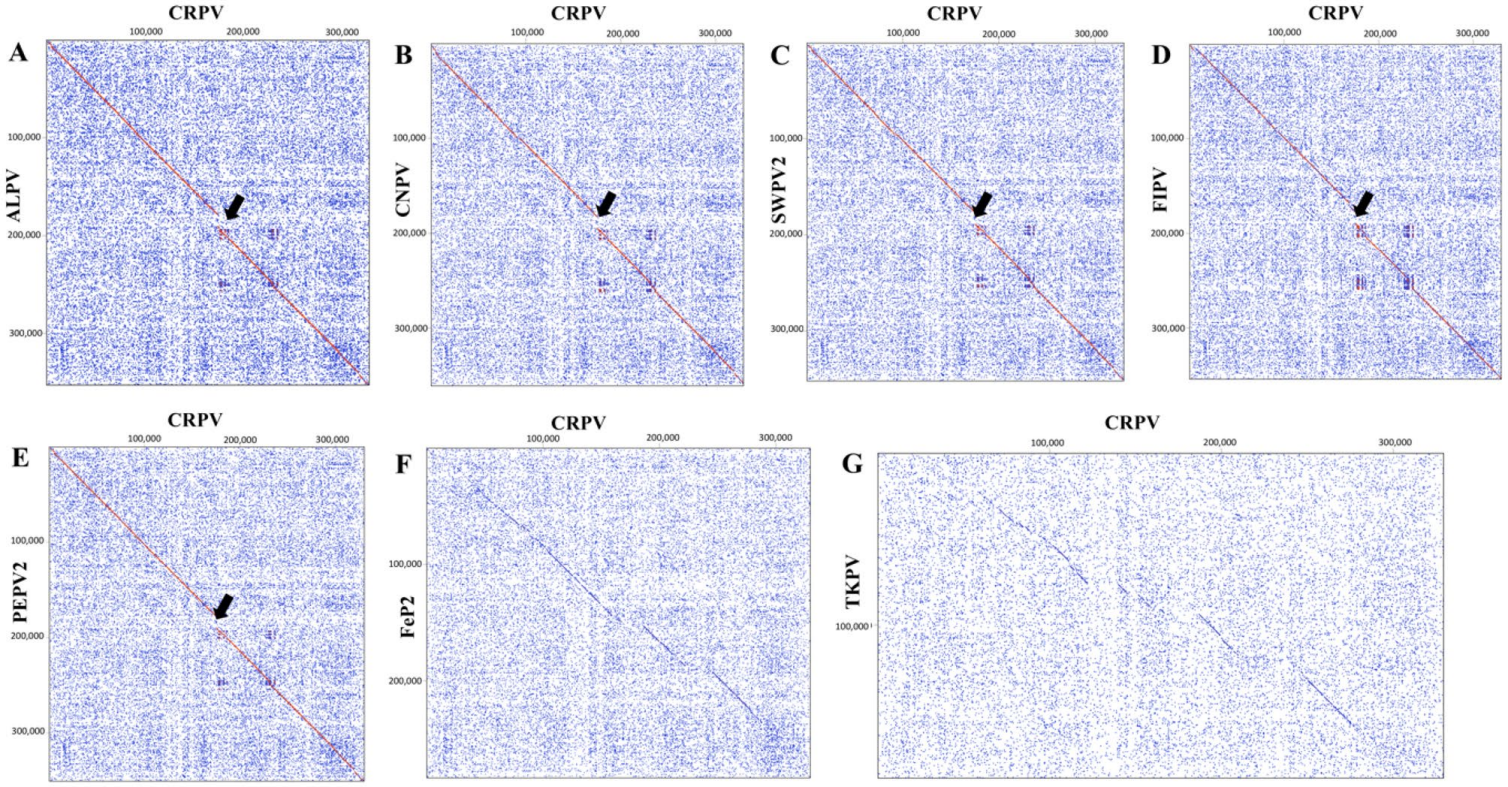
Dot plots of the CRPV genome (x-axis) versus other poxvirus genomes (y-axis). (**A**) CRPV versus ALPV, (**B**) CRPV versus CNPV, (**C**) CRPV versus SWPV2, (**D**) CRPV versus FIPV, (**E**) CRPV versus PEPV2, (**F**) CRPV versus FeP2 and (**G**) CRPV versus TKPV (refer to Table 3 for virus details and GenBank accession numbers). The Classic colour scheme was chosen in Geneious (version 22.1.1) for the dot plot lines according to the length of the match, from blue for short matches to red for matches over 100 bp long. Window size = 12.

Dot plot analyses were used to compare the CRPV genome with other selected avipoxviruses. The CRPV genome was highly syntenic with ALPV, CNPV, SWPV2, FIPV and PEPV2 (Fig. 2A–E); however, a difference in synteny was observed (Fig. 2A–E, highlighted as black arrows), mainly due to the absence of two large additional copies of variola B22R gene family proteins and a hypothetical protein coding gene covering approximately 16kbp. However, the CRPV genome demonstrated significant differences in the entire genome compared to other complete avipoxviruses including FeP2 and TKPV (Fig. 2F–G).

### Core/conserved ORFs

Similar to other chordopoxviruses (ChPVs) the CRPV genome contained 89 conserved core genes, which are involved in essential functions such as replication, transcription and virion assembly (Supplementary Table S1; highlighted with bold font). The number of conserved ChPV genes is considered to range between 83 and 90^3,9,28,29^, which is consistent with the findings in the CRPV genome. Among them, nine of the predicted ORFs (CRPV-143, -166, -193, -211 -244, -307, -317, -319 and -327) were truncated mostly with a single residue compared to CNPV, which may warrant further studies to determine whether they are expressed and functional. Based on a recent study by Carulei et al*.*^3^, we also searched for a further 47 genes that are conserved in avipoxviruses (Table 2). We predicted the CRPV genome would also contain these 47 conserved ORFs (Table 2), and eight of the genes (CRPV-049, -125, -131, -167, -173, -323, -324 and -341) were found to be truncated compared to a closely related canarypox virus (CNPV).

**Table 2:** 47 ORFs found to be uniquely conserved in the selected fully sequenced avian poxvirus genomes.

CRPV	ALPV2	FWPV	MPPV2	MPPV	SWPV2	SWPV1	CNPV	PEPV	FeP2	FGPV	TKPV	Function
48	33	16	44	34	28	24	32	19	19	11	001.1a	Ig-like domain
49	34	17	45	35	29	25	33	20	20	12	2	V-type Ig domain
57	40	20	53	41	34	28	38	24	24	17	5	C4L/C10L protein
58	41	21	54	42	35	29	39	25	25	18	6	GPCR
59	42	22	55	43	36	30	40	26	26	19	7	Ankyrin repeat
61	43	23	57	44	37	31	41	27	27	20	8	Ankyrin repeat
62	44	24	58	45	38	32	42	28	28	21	9	Ankyrin repeat
69	53	30	66	52	44	38	48	35	35	29	12	Alkaline phosphodiesterase
71	54	31	69	55	46	40	50	36	36	30	13	Ankyrin repeat
74	60	35	72	58	49	44	53	40	38	34	16	Hypothetical protein
77	62	37	74	60	51	46	55	41	39	36	17	Hypothetical protein
80	64	39	77	63	54	49	58	43	41	38	20	B-cell lymphoma 2 (Bcl-2)
81	65	40	78	64	55	50	59	44	42	39	21	Serpin
83	69	43	81	66	57	52	61	46	44	41	22	DNA ligase
84	70	44	82	67	58	53	62	47	45	42	23	Serpin family
85	71	46	83	68	59	54	63	48	46	43	24	Hydroxysteroid dehydrogenase
88	73	47	87	71	61	56	65	49	47	44	25	Semaphorin
91	76	48	92	75	64	59	68	50	48	45	26	GNS1/SUR4
98	82	54	103	83	72	66	76	56	54	51	32	mutT motif
120	96	65	-	-	83	78	88	67	65	64	40	Hypothetical protein
125	100	68	128	98	87	82	92	70	68	67	42	Hypothetical protein
128	102	70	130	100	89	84	94	72	70	69	44	T10-like protein
131	104	71	-	104	92	87	97	75	72	72	46	Hypothetical protein
137	109	75	140	110	98	92	103	78	77	76	50	N1R/p28
148	120	86	150	120	108	102	113	89	87	87	60	Thymidine kinase
154	126	91	156	126	113	107	118	95	93	93	65	Hypothetical protein
155	127	92	157	127	114	108	119	96	94	94	66	virion core protein
167	142	104	169	139	126	120	131	108	106	106	75	Hypothetical protein
168	143	105	170	140	127	121	132	109	107	107	76	Hypothetical protein
173	149	110	175	145	132	126	137	114	112	112	80	Hypothetical protein
176	152	113	178	148	135	129	140	117	115	115	83	Hypothetical protein
243	196	145	243	199	179	167	191	153	146	151	109	Hypothetical protein
252	203	151	253	209	187	175	199	159	153	157	113	Deoxycytidine kinase
323	255	190	331	274	250	237	264	203	195	204	140	A-type inclusion protein
324	256	191	333	275	251	238	265	204	196	205	141	A-type inclusion protein
329	262	196	339	280	256	243	270	210	202	211	144	Hypothetical protein
333	267	201	343	284	259	247	273	215	207	216	149	Hypothetical protein
334	269	203	344	285	260	248	274	216	208	217	150	Tyrosine kinase
336	271	205	346	287	262	250	276	218	210	219	151	Hypothetical protein
338	273	207	348	289	264	252	278	220	212	221	151.1a	Hypothetical protein
341	277	208	351	292	267	255	281	222	214	224	152	Hypothetical protein
345	280	211	355	296	271	259	285	225	216	227	153	Epidermal Growth Factor
346	281	212	356	297	272	260	286	226	217	228	154	Serine/threonine protein kinase
347	282	213	357	298	273	261	287	227	218	229	155	Hypothetical protein
350	284	214	361	300	275	263	289	228	219	230	156	Putative 13.7 kDa protein
357	293	219	370	308	282	272	296	234	226	238	161	Ankyrin repeat
374	312	232	394	327	290	283	304	248	238	251	164	Ankyrin repeat

The numbers in each column refer to the specific ORF in each respective genome.

### Multigene families

Avipoxviruses are the largest ChPVs and contain several, large, multigene families with immune related functions comprising up to 50% of the genome^3,9^. The copy numbers of each of the 14 multigene families identified in the CRPV genome compared with the other selected sequenced avian poxvirus genomes, including the recently characterised genomes of ALPV2, ALPV, MPPV2 and PEPV2 (Supplementary Table S2). CRPV has a relatively higher number of multigene families (156 gene copies) compared to the closely related avipoxviruses such as ALPV, CNPV and SWPV2 (total of 139, 137 and 124 gene copies, respectively). The copy number of ankyrin repeat, B22R, C4L/C10L, CC chemokine and TGF-β family genes were relatively higher in the CRPV genome compared to CNPV. However, the copy number of N1R/p28 and C-type lectin genes were significantly lower in the CRPV genome compared to CNPV.

### Evolutionary relationships of CRPV

**Figure 3:**
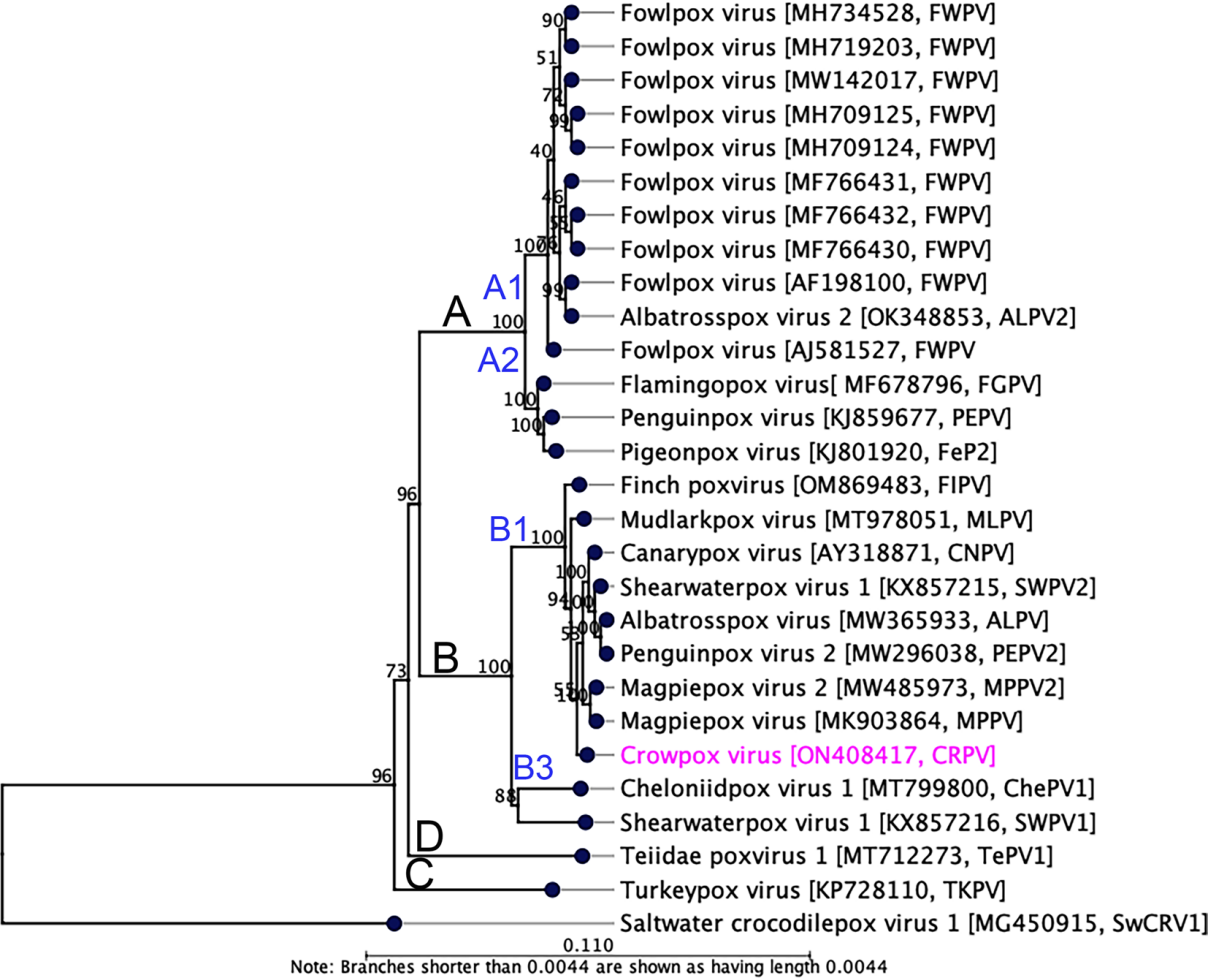
Phylogenetic relationships between CRPV and other chordopoxviruses. A maximum likelihood (ML) tree was constructed from multiple alignments of the concatenated amino acid sequences of the selected nine poxvirus core proteins using CLC Genomics Workbench (version 9.0.1). The numbers on the left show bootstrap values as percentages. The labels at branch tips refer to virus species, followed by GenBank accession numbers and abbreviated species names in parentheses. The position of CRPV is highlighted using pink text. Details of the poxviruses used in the phylogenetic tree are in Table 3. Saltwater crocodile poxvirus 1 (SwCRV1; MG450915)^30^ was used as an outgroup. Major clades and sub-clades are designated according to Gyuranecz et al*.* (2013)^25^.

Phylogenetic reconstruction using concatenated amino acid sequences of selected conserved ChPV genes provides clear evidence for the inclusion of CRPV in the genus *Avipoxvirus.* In the maximum likelihood (ML) tree (Fig. 3), CRPV was located within a subclade B1 encompassing avipoxviruses isolated from several passerine bird species, a wedge-tailed shearwater (*A. pacificus*), northern royal albatross (*Diomedea sanfordi*), and yellow-eyed penguin (*Megadyptes antipodes*) with 100% bootstrap support. In the sub-clade B1, FIPV is basal to known avipoxviruses, suggesting that all the avipoxviruses under this subclade evolved from the ancestral house finches (*Haemorhous mexicanus*) from where MLPV followed by CRPV viruses evolved, and later to the other avian hosts. Using the same set of concatenated protein sequences, we found that the maximum inter-lineage sequence identity values ranged from 98.7 to 97.9% among avipoxviruses under subclade B1, which reflected the phylogenetic position of this novel avipoxvirus sequenced from an Australian little crow, and further inferred that these viruses likely originated from a common ancestor. Furthermore, it was also evidenced that there were many avipoxviruses evolutionarily linked with crowpox virus sequenced in this study when we compared using partial nucleotide sequences of the DNA polymerase gene (Supplementary Fig. S1) and p4b gene (Supplementary Fig. S2). Among them, avipoxviruses isolated from an American crow (*Corvus brachyrhynchos*) and a canary (*Serinus canaria*) in Chile^25^, Australian magpies^17,18^, a gray-crowned rosy finch (*Leucosticte tephrocotis*) and a Swainson's thrush (*Catharus ustulatus*) in the USA^25^ were the closest evolutionary link with the CRPV isolated in this study.

### Evidence of poxvirus particles in cutaneous pox tissue

**Figure 4:**
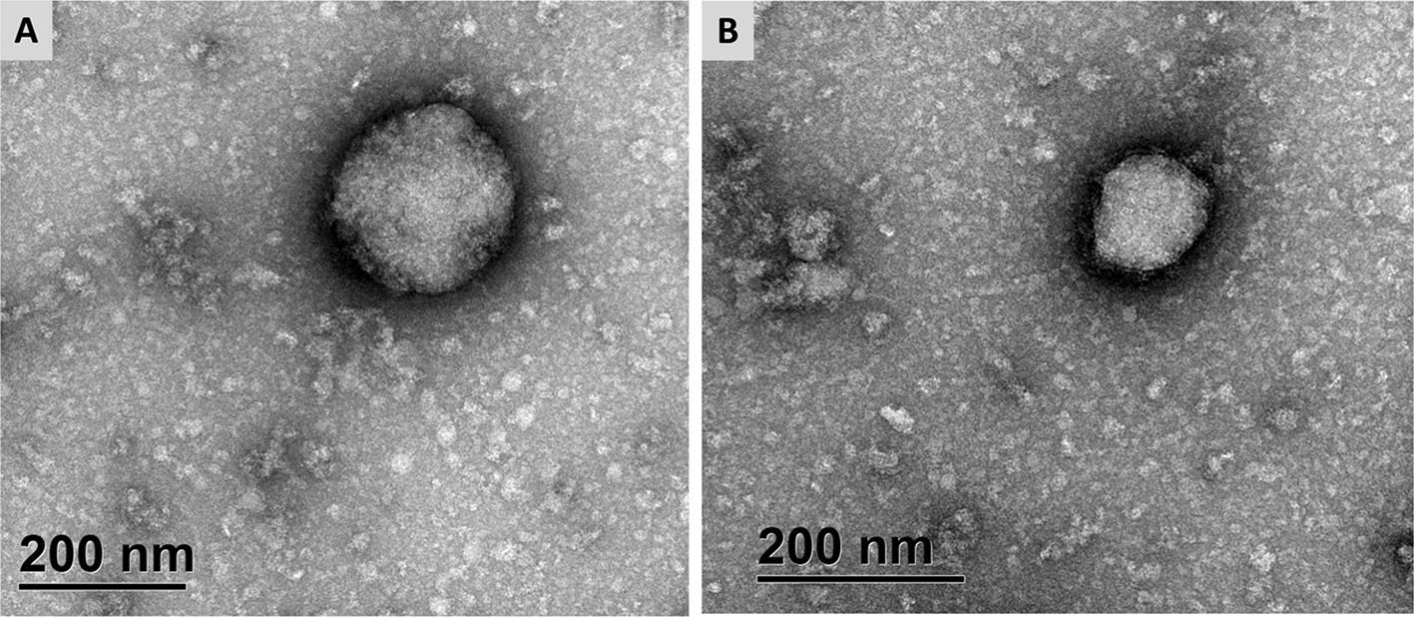
Transmission electron microscopic analysis of negatively stained cutaneous tissue sourced from a passerine bird, Australian little crow. Crowpox virus particles showed the presence of intracellular mature virion (**A**) and immature virion (**B**).

Using transmission electron microscopy (TEM) analysis, crowpox virus particles were identified in the sample sourced from the Australian little crow. It showed the presence of intracellular mature virion (IMV) that was brick-ovoid shaped with regular spaced thread-like ridges comprising the exposed surface and was approximately 250 × 230 nm (Fig. 4A), and immature virion was brick shaped, measuring approximately 135 × 125 nm in diameter (Fig. 4B).

## Discussion

This paper documents a novel avipoxvirus, crowpox virus (CRPV), with the complete genome sequence obtained directly from naturally occurring pox lesions in an Australian little crow. Applying various approaches for genomic comparison and transmission electron microscopic analysis, the CRPV genome was shown to be architecturally consistent with other avipoxviruses in terms of genome size, AT content and predicted ORFs; however, it was distinct from other avipoxvirus genomes in multiple ways. Overall, the DNA sequence of CRPV was significantly different from other avipoxviruses but had the closest similarity with the pathogenic avipoxvirus, albatrosspox virus (ALPV), followed by penguinpox virus 2 (PEPV2; 84.3%), shearwaterpox virus 2 (SWPV2; 83.4), canarypox virus (CNPV; 83.3%), and magpiepox virus 2 (MPPV2; 82.6%). The novel CRPV genome contained 47 predicted genes that are not found in any other poxvirus, as well as several ORFs that were so truncated/fragmented as to probably cause them to be non-functional. Overall, the CRPV was sufficiently genetically different to other previously classified avipoxviruses to be considered as a distinct new virus species under the genus *Avipoxvirus*.

Phylogenetic tree analysis demonstrated that the subclade B1 consisting of avipoxviruses isolated from several passerine bird species including this Australian little crow, as well as seabirds including a wedge-tailed shearwater, a northern royal albatross, and a yellow-eyed penguin, supports evidence that this CRPV most likely originated from avipoxvirus isolated from other birds in the order Passeriformes (Fig. 3). The basal position of a recently sequenced avipoxvirus from house finches in the USA (subclade B1, Fig. 3), from where mudlarkpox virus (MLPV) followed by CRPV viruses have likely evolved provides evidence that the evolution of avipoxviruses in Passeriformes is not well understood, given the divergent geographical distribution of the host species. It is possible that Australian passerine birds may be host to many as yet undiscovered avipoxviruses.

Much is still unknown about the host spectrum and epidemiology of poxviruses, in particular for Australian avifauna. Although there is some evidence that poxviruses infecting Australian wild birds including magpies are transmissible to other magpies, but not to chickens, turkeys, pigeons, or canaries after experimental inoculation^31,32^, their mode of transmission in Australian avifauna is not well understood. Previous studies suggest that avipoxviruses can be transmitted between birds in several ways: (1) via direct contact with infected birds through broken skin; (2) through contact between skin breaches and contaminated objects including perches; (3) by aerosol transmission^4,33^, and (4) via haematophagous arthropods including mosquitoes, which are efficient mechanical vectors through contaminated mouthparts^34,35^. These factors may indicate a potential scenario for crowpox virus transmission that merits further attention. At an individual level, poxvirus infections in wild birds can cause primary disease that may be severe in some cases, leading to decreased foraging and mobility. In affected birds, avipoxvirus infection can lead to two different forms of disease. The most common disease is characterised by a proliferative ‘wart-like’ lesions that are commonly restricted to the eyes, beak or unfeathered skin of the body (so-called ‘dry’ pox), in which secondary bacterial and fungal infections may aggravate the birds' condition. The second form of poxviral infection is the ‘wet’ or ‘diphtheritic’ form, characterised by lesions on the mucous membranes of the upper alimentary and respiratory tracts^2,36^. Avipoxvirus infection in bird can also reduce ability to care for young, and affect vision and/or feeding ability, making them prone to predation, and significantly affecting welfare^2,36,37^, but in some cases, birds are likely sub clinically infected. The repeated occurrence of avian family or order-specific grouping within certain avipoxvirus clades indicates a marked role of host adaptation, while the sharing of poxvirus species within prey-predator systems (e.g., pigeonpox in raptors)^25^ indicates the potential for cross-species infection and limited host adaptation^25^. At a population level, these may have serious implications, especially for endangered or endemic species, and hence further studies into the evolution of avipoxviruses in non-model hosts warrants further investigation.

## Conclusions

The novel complete genome sequence of CRPV reported here has enhanced the genomic information for the *Avipoxvirus* genus, contributing to our understanding of the avipoxviruses more generally, as well as tracking poxvirus evolution in a non-model avian species. By assessing the sequence similarity between CRPV and other avipoxviruses, we concluded that the CRPV complete genome described should be considered a separate avipoxvirus species. Additional investigations will be required to better understand relevant host–pathogen dynamics including routes of transmission and factors leading to infection, associated pathology, and disease prevalence.

## Methods

### Sampling, ethical consideration and extraction of DNA

Samples were obtained from a little crow (*Corvus bennetti*) that was euthanised by inhalational general anaesthesia with isoflurane (IsoFlo, Zoetis Australia Pty Ltd) in oxygen followed by intravenous injection of pentabarbitone sodium (Lethabarb Euthanasia Injection, Virbac Australia Pty Ltd) due to untreatable septic arthritis secondary to proliferative pox lesions by a registered veterinarian at The Unusual Pet Vets, Frankston, Victoria. Animal sampling (ID: 122740) was carried out by the attending veterinarian for the investigation of crusty pox lesions affecting the legs and eyes. Collected samples were stored at − 20 °C until further processing. For DNA extraction, the crusty pox lesion material was aseptically dissected and mechanically homogenised in lysis buffer using disposable tissue grinder pestles and transferred into a 1.5 mL microcentrifuge tube (Eppendorf). Total genomic DNA was isolated according to the established methods^38,39^ using a ReliaPrep gDNA Tissue Miniprep System (Promega, USA).

### Library construction and sequencing

A total of 250 ng of extracted genomic DNA was used to prepare the library using the protocol adapted previously using the Illumina DNA Prep (Illumina, San Diego, CA, USA)^40^. The quality and quantity of the prepared library was assessed using an Agilent Tape Station (Agilent Technologies) by the Genomic Platform, La Trobe University. The prepared library was sequenced with the sequencing reads length of 150-bp paired-end on Illumina^®^ NovaSeq platform according to the manufacturer's instructions through the Australian Genome Research Facility, Melbourne.

### Genome assembly and annotation

The resulting 39.8 million raw sequence reads were used to assemble the complete genome of CRPV, using CLC Genomics Workbench (version 9.0.1, CLC bio, a QIAGEN Company, Prismet, Aarhus C, Denmark) and Geneious Prime^®^ (version 2022.1.1, Biomatters, New Zealand), as described previously^16,17,20,26,41^. Briefly, the sequences were processed to remove Illumina adapters, low quality reads and ambiguous bases. Trimmed sequence reads were mapped against the chicken genome (*Gallus gallus*, GenBank accession number NC_006088.5) to remove potential host DNA contamination. In addition, reads were further mapped to the *Escherichia coli* bacterial genomic sequence (GenBank accession no. U00096) to remove possible bacterial contamination. A total of 30.2 million cleaned and unmapped reads were used as input data for de novo assembly using CLC Genomics Workbench (version 9.0.1). This resulted in the generation of a 328,768 bp genome with an average coverage of 1182.51x. The genome was annotated according to the previously published protocol using Geneious software (version 2022.1.1). Open reading frames (ORFs) longer than 30 amino acids, with a methionine start codon (ATG) and minimal overlap with other ORFs (not exceeding 50% of one of the genes), were selected and annotated. ORFs shorter than 30 amino acids that had been previously annotated in other poxvirus genomes were also included. Similarity BLAST searches were performed on the predicted ORFs and were annotated as potential genes if predicted ORFs showed significant sequence similarity to known viral or cellular genes (BLAST E value ≤ e^−5^)^27^. The direct tandem repeats were detected using the Tandem Repeats Finders^42^.

To predict the function of putative unique ORFs identified in this study, the derived protein sequence of each ORF was searched using multiple applications to identify conserved domains or motifs. Transmembrane helices were searched using the TMHMM package (version 2.0)^43^ and TMpred^44^. Additionally, searches for conserved secondary structure (HHpred)^45^ and protein homologs were conducted using Phyre2^46^ and SWISS-MODEL^47^.

### Comparative genomics

Genomic features of the newly sequenced CRPV were visualised using Geneious Prime^®^ (version 2022.1.1). Sequence similarity percentages between CRPV and representative ChPV complete genome sequences were determined using tools available in Geneious (version 2022.1.1). Dot plots were created based on the EMBOSS dottup program in Geneious software, with word size = 12^48^.

### Phylogenetic analyses

Phylogenetic analyses were performed using the CRPV genome sequence determined in this study, together with other selected ChPV genome sequences available in GenBank (Table 3). The amino acid sequences of nine poxvirus core proteins (RNA polymerase subunit RPO132, RNA polymerase subunit RPO147, mRNA capping enzyme large subunit, RNA polymerase-associated protein RAP94, virion core protein P4a, virion core protein P4b, early transcription factor large subunit VETFL, NTPase, and DNA polymerase) were concatenated and aligned using MAFTT (version 7.450) with the G-INS-i (gap open penalty 1.53; offset value 0.123) algorithm implemented in Geneious Prime^®^ (version 2022.1.1, Biomatters, New Zealand). Nucleotide sequences of the partial DNA polymerase and partial p4b genes, as well as concatenated amino acid sequences of the selected nine poxvirus core proteins, were aligned as described previously^19^ using the MAFTT L-INS-I algorithm implemented in Geneious Prime^®^ (version 2022.1.1) (version 7.388)^49^. To determine the best-fit model to construct phylogenetic analyses, a model test was performed using CLC Genomics Workbench (version 9.0.1), which favoured a general-time-reversible model with gamma distribution rate variation and a proportion of invariable sites (GTR + G + I). Phylogenetic analyses for nucleotide sequences were performed under the GTR substitution model, but the WAG substitution model was chosen for concatenated amino acid sequences with 1000 bootstrap replicates in CLC Genomic Workbench (version 9.0.1).

**Table 3:** Related poxvirus genome sequences used in further analysis of CRPV.

Virus	Abbreviation	Year of isolation	GenBank accession number	References
Crowpox virus	CRPV	2021	ON408417	This study
Albatrosspox virus 2	ALPV2	1997	OK348853	^21^
Albatrosspox virus	ALPV	1997	MW365933	^22^
Canarypox virus	CNPV	1948	AY318871	^8^
Canarypox virus	CNPV	2015	MG760432	^50^
Cheloniidpox virus 1	ChePV1	2018	MT799800	^51^
Fowlpox virus	FWPV	2012, 2000*, 2010*, 2015, 2016, 2018*, 2011# 2018	MW142017, AF198100*, AJ581527*, MH734528, MH719203, MF766430-32, MH709124-25*, MG702259#, OK558608-09	^11,13–15,52^
Flamingopox virus	FGPV	2008	MF678796	^3^
Finch poxvirus	FIPV	2021	OM869483	^23^
Magpiepox virus	MPPV	2018	MK903864	^17^
Magpiepox virus 2	MPPV2	1956	MW485973	^18^
Mudlarkpox virus	MLPV	2019	MT978051	^19^
Penguinpox virus	PEPV	1992	KJ859677	^9^
Penguinpox virus 2	PEPV2	1997	MW296038	^20^
Pigeonpox virus	FeP2	1992	KJ801920	^9^
Saltwater crocodilepox virus 1	SwCRV1	2017	MG450915	^30,38^
Shearwaterpox virus 1	SWPV1	2015	KX857216	^16^
Shearwaterpox virus 2	SWPV2	2015	KX857215	^16^
Turkeypox virus	TKPV	2011	NC_028238	^10^
Teiidae poxvirus 1	TePV1	2019	MT712273	^53^

* = the year of submission to GenBank is reported, # = unpublished.

### Transmission electron microscopy

Cutaneous pox lesions were suspended 1:10 in phosphate-buffered saline (PBS), homogenised, clarified and adsorbed onto 400-mesh copper EM grids, before staining and imaging on a JEOL JEM-2100 transmission electron microscope as previously described^26,30^.

### Institutional review board statement

The animal handling and procedures were conducted in accordance with ARRIVE guidelines for experimental procedures. Briefly, samples were obtained from a little crow (*Corvus bennetti)* that was euthanatised due to untreatable septic arthritis secondary to proliferative pox lesions by a registered veterinarian at The Unusual Pet Vets, Frankston, Victoria. The animal was necropsied by the same registered veterinarian for routine diagnostic purposes. All other methods were performed in accordance with the standard guidelines and regulations for PC2 laboratory. The Animal Ethics Committee at La Trobe University was informed that findings from the diagnostic material were to be used in a publication, and a formal waiver of ethics approval was granted.

## Supplementary Information

Supplementary Information.

## Data Availability

The complete genome sequence and associated datasets generated during this study were deposited in GenBank under the accession number ON408417. Raw sequencing data from this study has been deposited in the NCBI Sequence Read Achieve (SRA) under the accession number SRR19117728 (BioProject ID: PRJNA835616, BioSample accessions: SAMN28105687) (http://www.ncbi.nlm.nih.gov/sra/).
