# Supplementary material for: Molecular characterisation of a novel pathogenic avipoxvirus from an Australian little crow (Corvus bennetti) directly from the clinical sample
Source: Sci Rep. 2022 Sep 5;12:15053. doi: 10.1038/s41598-022-19480-2 (PMC9445014; doi:10.1038/s41598-022-19480-2)
Supplement: Supplementary file 1 — Supplementary Information. [file 41598_2022_19480_MOESM1_ESM.pdf]

Supplementary File

Molecular characterisation of a novel pathogenic avipoxvirus from an Australian little crow (*Corvus bennetti*) directly from a clinical sample

Subir Sarker [1*] and Michelle Sutherland[2]

[1]Department of Physiology, Anatomy and Microbiology, School of Life Sciences, La Trobe University, Melbourne, VIC 3086, Australia.
[2] The Unusual Pet Vets, 210 Karingal Drive, Frankston, Vic 3199, Australia

*Address for correspondence: Dr. Subir Sarker, Department of Physiology, Anatomy and Microbiology, School of Life Sciences, La Trobe University, Melbourne, VIC 3086, Australia; email: S.Sarker@latrobe.edu.au; phone: +61 3 9479 2317; fax: +61 3 9479 1222.

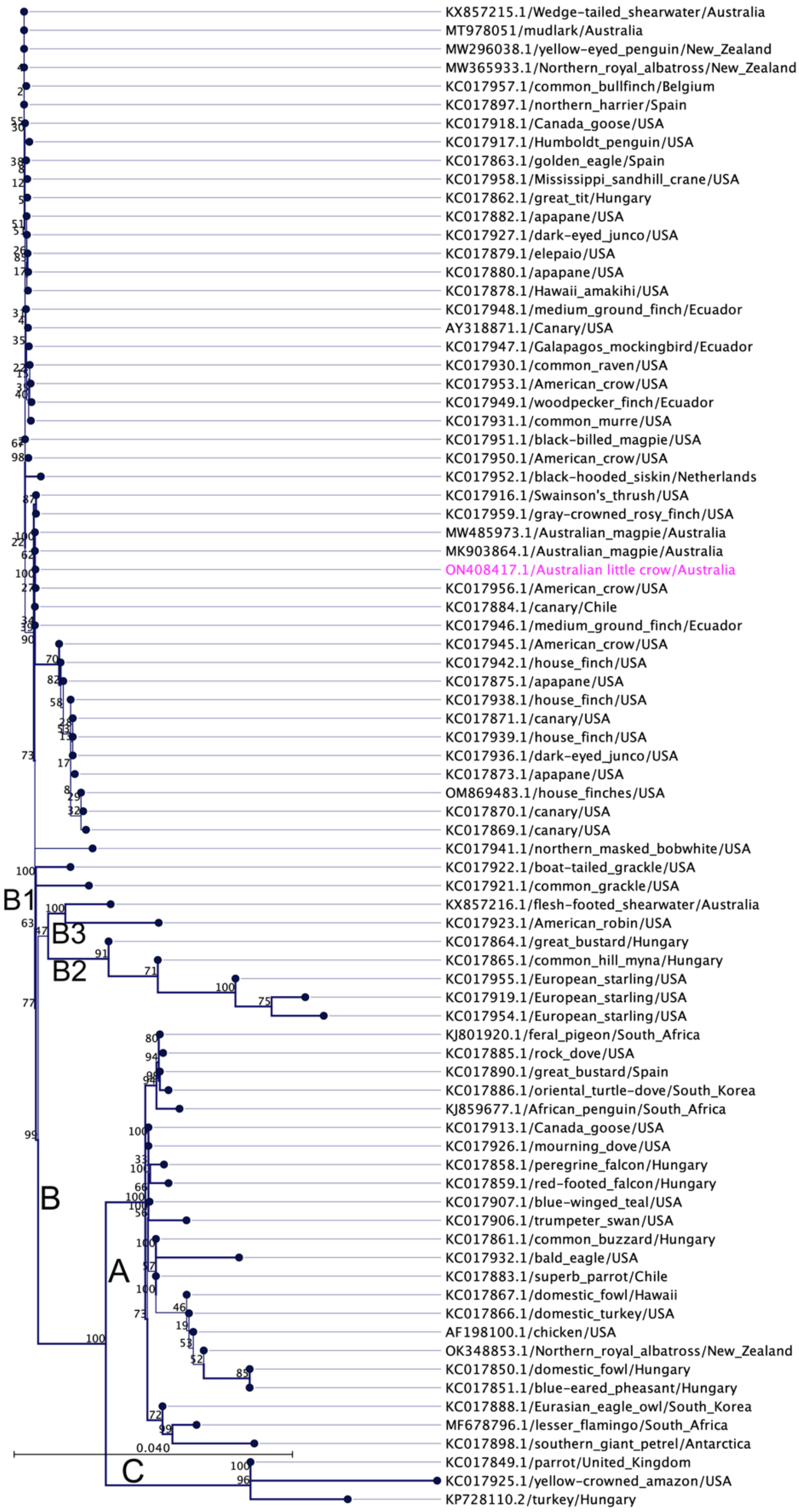

Figure S1: Maximum likelihood (ML) phylogenetic tree from partial nucleotide sequences of the DNA polymerase gene of selected avipoxviruses. Labels at branch tips refer to GenBank accession number/species/country of origin. The numbers on the left show bootstrap values as percentages. The position of CRPV is highlighted using pink text. The ML tree is displayed as a phylogram. The bootstrap value assigned to a node in the output tree is the percentage (0-100) of the bootstrap resamples which resulted in a tree containing the same subtree as that rooted at the node. Major clades and sub-clades are designated according to Gyuranecz *et al* (2013).

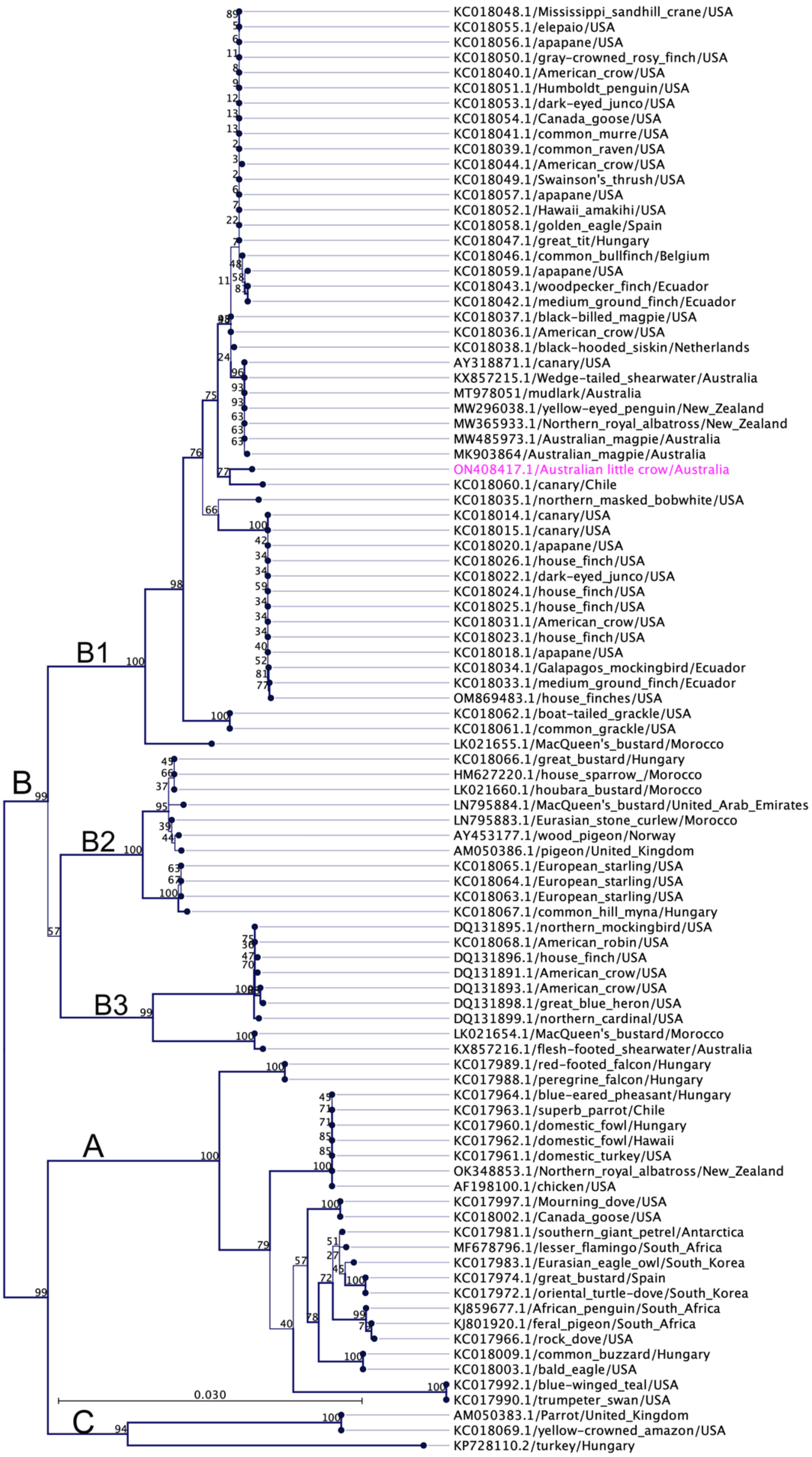

Figure S2: Maximum likelihood (ML) phylogenetic tree from partial nucleotide sequences of the P4b gene of selected avipoxviruses. Labels at branch tips refer to GenBank accession number/species/country of origin. The numbers on the left show bootstrap values as percentages. The position of CRPV is highlighted using pink text. The ML tree is displayed as a phylogram. The bootstrap value assigned to a node in the output tree is the percentage (0-100) of the bootstrap resamples which resulted in a tree containing the same subtree as that rooted at the node. Major clades and sub-clades are designated according to Gyuranecz *et al* (2013).

Supplementary Table S1. Crowpox virus (CRPV) genome annotations and comparative analysis of ORFs

CRPV Synteny	CRPV Genome Coordinates	CNPV Synteny	CNPV AA size	CRPV AA size	Best BLAST hits	CRPV AA Identity (%) compared to avipoxviruses	Notes
CRPV-001	631-1107	CNPV002	171	158	CNPV002 hypothetical protein	60.6	
CRPV-002	1761-1024	CNPV004	514	245	CNPV004 ankyrin repeat protein	55.1	
CRPV-003	2456-1788	CNPV004	514	222	CNPV004 ankyrin repeat protein	71.8	
CRPV-004	2699-3367	CNPV005	222	222	MPPV-005 conserved hypothetical protein	97.3	
CRPV-005	3327-3908	CNPV006	182	193	SWPV2-309 conserved hypothetical protein	84.5	
CRPV-006	3930-4082			50			hypothetical protein, unique to CRPV, containing a transmembrane helix
CRPV-007	4321-4202			39			hypothetical protein, unique to CRPV, containing a transmembrane helix
CRPV-008	6354-4321	CNPV009	688	677	CNPV009 ankyrin repeat protein	98.1	
CRPV-009	6600-6499			33			hypothetical protein, unique to CRPV
CRPV-010	6893-6729	CNPV010	734	54	CNPV010 ankyrin repeat protein	90.6	
CRPV-011	6915-7019			34			hypothetical protein, unique to CRPV
CRPV-012	7248-7111	CNPV010	734	45	CNPV010 ankyrin repeat protein	82.2	
CRPV-013	7453-7572			39			hypothetical protein, unique to CRPV
CRPV-014	7781-7614	CNPV010	734	55	CNPV010 ankyrin repeat protein	50.0	
CRPV-015	7890-7756			44			hypothetical protein, unique to CRPV

CRPV Synteny	CRPV Genome Coordinates	CNPV Synteny	CNPV AA size	CRPV AA size	Best BLAST hits	CRPV AA Identity (%) compared to avipoxviruses	Notes
CRPV-016	8077-7916			53			hypothetical protein, unique to CRPV, containing a transmembrane helix
CRPV-017	8399-8235			54			hypothetical protein, unique to CRPV, containing a transmembrane helix
CRPV-018	9880-8318	CNPV011	586	520	CNPV011 ankyrin repeat protein	68.5	
CRPV-019	10060-9956			34			hypothetical protein, unique to CRPV
CRPV-020	10339-10163			58			hypothetical protein, unique to CRPV
CRPV-021	10342-10617	CNPV012	189	91	CNPV012 hypothetical protein	78.9	
CRPV-022	11330-10824	CNPV013	168	168	MPPV013 conserved hypothetical protein	90.5	
CRPV-023	11915-11457			152	FGPVKD09 N1R/p28 family protein	64.4	
CRPV-024	13480-12059	CNPV014	490	473	MPPV014 Ig-like domain protein	89.3	
CRPV-025	13651-15237	CNPV015	528	528	CNPV015 ankyrin repeat protein	85.1	
CRPV-026	15298-15804	CNPV016	168	168	CNPV016 C-type lectin protein	93.5	
CRPV-027	15908-17344	CNPV017	486	478	CNPV017 ankyrin repeat protein	96.2	
CRPV-028	18019-17447	CNPV018	191	190	CNPV018 IL-10-like protein	78.7	
CRPV-029	19440-18130	CNPV019	436	436	CNPV019 ankyrin repeat protein	91.5	
CRPV-030	19621-20880	CNPV020	419	419	CNPV020 ankyrin repeat protein	96.4	
CRPV-031	21111-21010	CNPV021	535	33	SWPV2-017 ankyrin repeat protein	87.9	
CRPV-032	21782-21234	CNPV021	535	182	CNPV021 ankyrin repeat protein	88.7	
CRPV-033	22498-22019	CNPV021	535	159	CNPV021 ankyrin repeat protein	88.0	

CRPV Synteny	CRPV Genome Coordinates	CNPV Synteny	CNPV AA size	CRPV AA size	Best BLAST hits	CRPV AA Identity (%) compared to avipoxviruses	Notes
CRPV-034	23615-22539	CNPV022	358	358	CNPV022 putative serpin	95.5	
CRPV-035	24108-23851	CNPV023	427	85	CNPV023 vaccinia C4L/C10L-like protein	71.9	
CRPV-036	24507-24358	CNPV023	427	49	CNPV023 vaccinia C4L/C10L-like protein	73.1	
CRPV-037	24819-24529	CNPV023	427	96	CNPV023 vaccinia C4L/C10L-like protein	92.1	
CRPV-038	25083-25619	CNPV024	178	178	CNPV024 hypothetical protein	89.3	
CRPV-039	26730-25828	CNPV025	300	300	CNPV025 alpha-SNAP-like protein	91.0	
CRPV-040	27970-26822	CNPV026	382	382	CNPV026 ankyrin repeat protein	94.2	
CRPV-041	29919-28039	CNPV027	626	626	CNPV027 ankyrin repeat protein	91.7	
CRPV-042	31141-30053	CNPV028	362	362	CNPV028 ankyrin repeat protein	94.8	
CRPV-043	31203-31087			38			hypothetical protein, unique to CRPV
CRPV-044	31678-31250	CNPV029	142	142	CNPV029 C-type lectin-like protein	99.3	
CRPV-045	32785-31763	CNPV030	340	340	CNPV030 ankyrin repeat protein	96.2	
CRPV-046	33025-33387	CNPV031	119	120	CNPV031 hypothetical protein	95.8	
CRPV-047	33431-33535			34			hypothetical protein, unique to CRPV
**CRPV-048**	**34342-33614**	**CNPV032**	**242**	**242**	**MPPV-034 Ig-like domain putative IFN-gamma binding protein**	**92.6**	
**CRPV-049**	**35144-34416**	**CNPV033**	**246**	**242**	**MPPV-035 Ig-like domain protein**	**87.3**	
CRPV-050	35167-35277			36			hypothetical protein, unique to CRPV, containing a transmembrane helix
CRPV-051	37214-35238	CNPV034	659	658	CNPV034 ankyrin repeat protein	91.2	
CRPV-052	37103-37405			100			hypothetical protein, unique to CRPV, containing three

CRPV Synteny	CRPV Genome Coordinates	CNPV Synteny	CNPV AA size	CRPV AA size	Best BLAST hits	CRPV AA Identity (%) compared to avipoxviruses	Notes
							transmembrane helices
CRPV-053	37933-37529	CNPV035	134	134	MPPV-038 C-type lectin-like protein	88.1	
CRPV-054	38174-37977	CNPV036	95	65	CNPV036 conserved hypothetical protein	88.4	
CRPV-055	38212-38409			65	CNPV037 conserved hypothetical protein	98.3	
CRPV-056	38585-38755	CNPV037	179	56	MPPV-040 conserved hypothetical protein	96.4	
**CRPV-057**	**40025-38760**	**CNPV038**	**413**	**421**	**MPPV-041 vaccinia C4L/C10L-like protein**	**95.0**	
**CRPV-058**	**40143-41129**	**CNPV039**	**327**	**328**	**CNPV039 G protein-coupled receptor-like protein**	**91.8**	
**CRPV-059**	**42922-41147**	**CNPV040**	**591**	**591**	**MPPV-043 ankyrin repeat protein**	**92.2**	
CRPV-060	42902-43048			48			hypothetical protein, unique to CRPV, containing a transmembrane helix
**CRPV-061**	**44287-42995**	**CNPV041**	**430**	**430**	**CNPV041 ankyrin repeat protein**	**96.7**	
**CRPV-062**	**46153-44336**	**CNPV042**	**605**	**605**	**SWPV2-038 ankyrin repeat protein**	**93.9**	
CRPV-063	46862-46257	CNPV043	201	201	CNPV043 hypothetical protein	96.5	
CRPV-064	48346-46904	CNPV044	480	480	MPPV-047 ankyrin repeat protein	94.0	
CRPV-065	48679-49665	CNPV045	332	328	MPPV-048 G protein-coupled receptor-like protein	91.9	
CRPV-066	49709-49831			40			hypothetical protein, unique to CRPV
CRPV-067	50477-50608			43			hypothetical protein, unique to CRPV
CRPV-068	51330-50956	CNPV047	124	124	CNPV047 conserved hypothetical protein	96.8	
**CRPV-069**	**53934-51502**	**CNPV048**	**801**	**810**	**CNPV048 alkaline phosphodiesterase-like protein**	**91.0**	

CRPV Synteny	CRPV Genome Coordinates	CNPV Synteny	CNPV AA size	CRPV AA size	Best BLAST hits	CRPV AA Identity (%) compared to avipoxviruses	Notes
CRPV-070	54471-54019	CNPV049	150	150	CNPV049 hypothetical protein	96.7	
**CRPV-071**	**55580-54513**	**CNPV050**	**352**	**355**	**MPPV-055 ankyrin repeat protein**	**92.9**	
CRPV-072	56820-55627	CNPV051	401	397	CNPV051 DNase II-like protein	93.1	
CRPV-073	57366-56854	CNPV052	171	170	CNPV052 C-type lectin-like protein	92.9	
**CRPV-074**	**57950-57510**	**CNPV053**	**146**	**146**	**MPPV-058 conserved hypothetical protein**	**98.0**	
CRPV-075	58365-57943	CNPV054	140	140	CNPV054 hypothetical protein	90.0	
CRPV-076	58350-58457			35			hypothetical protein, unique to CRPV, containing a transmembrane helix
**CRPV-077**	**58908-58417**	**CNPV055**	**163**	**163**	**MPPV-060 conserved hypothetical protein**	**95.1**	
CRPV-078	59342-58905	CNPV057	306	145	CNPV056 dUTPase	97.2	
CRPV-079	60280-59369	CNPV056	145	303	CNPV057 putative serpin	95.1	
**CRPV-080**	**60852-60325**	**CNPV058**	**175**	**175**	**CNPV058 bcl-2 like protein**	**89.1**	
**CRPV-081**	**61928-60912**	**CNPV059**	**338**	**338**	**CNPV059 putative serpin**	**97.0**	
CRPV-082	62809-61997	CNPV060	316	270	PEPV2-059 hypothetical protein	96.3	
**CRPV-083**	**64595-62898**	**CNPV061**	**565**	**565**	**CNPV061 DNA ligase**	**98.2**	
**CRPV-084**	**65689-64634**	**CNPV062**	**350**	**351**	**CNPV062 putative serpin**	**95.4**	
**CRPV-085**	**66836-65760**	**CNPV063**	**358**	**358**	**CNPV063 hydroxysteroid dehydrogenase-like protein**	**94.1**	
CRPV-086	67358-66897	CNPV064	282	153	CNPV064 TGF-beta-like protein	94.1	
CRPV-087	67731-67618	CNPV064	282	37	SWPV2-060 TGF-beta-like protein	78.4	
**CRPV-088**	**69572-67821**	**CNPV065**	**583**	**583**	**CNPV065 semaphorin-like protein**	**92.5**	
CRPV-089	70117-69677	CNPV066	405	146	CNPV066 hypothetical protein	85.0	
CRPV-090	70384-70211	CNPV067	57	57	CNPV067 hypothetical protein	94.7	

CRPV Synteny	CRPV Genome Coordinates	CNPV Synteny	CNPV AA size	CRPV AA size	Best BLAST hits	CRPV AA Identity (%) compared to avipoxviruses	Notes
**CRPV-091**	**70547-71320**	**CNPV068**	**257**	**257**	**MPPV-075 GNS1/SUR4-like protein**	**98.1**	
**CRPV-092**	**71413-71880**	**CNPV069**	**155**	**155**	**CNPV069 late transcription factor VLTF-2**	**100.0**	
**CRPV-093**	**71897-73552**	**CNPV070**	**551**	**551**	**CNPV070 putative rifampicin resistance protein, IMV assembly**	**98.6**	
**CRPV-094**	**73584-74453**	**CNPV071**	**289**	**289**	**CNPV071 mRNA capping enzyme small subunit**	**99.0**	
CRPV-095	74490-75350	CNPV072	312	286	PEPV2-072 cc chemokine-like protein	38.8	
**CRPV-096**	**75390-77297**	**CNPV074**	**635**	**635**	**CNPV074 NPH-I, transcription termination factor**	**97.6**	
**CRPV-097**	**77975-77280**	**CNPV075**	**230**	**231**	**CNPV075 mutT motif putative gene expression regulator**	**99.1**	
**CRPV-098**	**78657-77959**	**CNPV076**	**232**	**232**	**CNPV076 mutT motif**	**99.6**	
CRPV-099	78629-78766			45			hypothetical protein, unique to CRPV, containing a transmembrane helix
CRPV-100	78879-78989			36			hypothetical protein, unique to CRPV, containing a transmembrane helix
CRPV-101	79255-79061	CNPV077	78	64	CNPV077 hypothetical protein	100.0	
CRPV-102	79379-79483			34	PEPV2-077 hypothetical protein	91.2	
CRPV-103	80153-80031			40	ALPV-081 hypothetical protein	87.8	
**CRPV-104**	**80751-80269**	**CNPV077**	**78**	**160**	**CNPV078 RNA polymerase subunit RPO18**	**99.4**	
CRPV-105	81887-81099	CNPV078	160	262	MPPV-085 Ig-like domain protein	76.0	
CRPV-106	81929-82072			47			hypothetical protein, unique to CRPV,

CRPV Synteny	CRPV Genome Coordinates	CNPV Synteny	CNPV AA size	CRPV AA size	Best BLAST hits	CRPV AA Identity (%) compared to avipoxviruses	Notes
							containing a transmembrane helix
**CRPV-107**	**83945-82044**	**CNPV080**	**633**	**633**	**CNPV080 early transcription factor small subunit VETFS**	**99.4**	
CRPV-108	85174-84158	CNPV081	333	338	ChePV1-078 Ig-like domain protein	78.2	
CRPV-109	85402-85292			36			hypothetical protein, unique to CRPV
CRPV-110	87879-85495	CNPV082	794	794	CNPV082 NTPase, DNA replication	98.4	
CRPV-111	88588-87995			197	viral CC-type chemokine [FIPV]	90.1	
**CRPV-112**	**89262-88606**	**CNPV084**	**218**	**218**	**uracil DNA glycosylase [FIPV]**	**95.4**	
CRPV-113	89588-89298	CNPV085	403	96	ALPV-089 putative RNA phosphatase	73.2	
CRPV-114	89706-89578			42	MLPV082 putative RNA phosphatase	95.2	
CRPV-115	90941-89754			395	hypothetical protein [PEPV2]	98.2	
CRPV-116	91027-91392	CNPV086	117	121	tnfr-like protein [PEPV2]	99.2	
CRPV-117	92673-91483	CNPV217	330	396	N1R/p28-like protein [MLPV]	81.1	
CRPV-118	92835-92963			42	GSH peroxidase [FIPV]	88.1	
CRPV-119	93036-93431	CNPV087	198	131	SWPV2-082 putative glutathione peroxidase	100.0	
**CRPV-120**	**93456-93758**	**CNPV088**	**100**	**100**	**SWPV2-083 conserved hypothetical protein**	**100.0**	
CRPV-121	94242-93763	CNPV089	159	159	hypothetical protein CNPV089	93.7	
CRPV-122	94607-94497			36			hypothetical protein, unique to CRPV
CRPV-123	94947-94696	CNPV091	83	83	CNPV091 HT motif protein	96.4	
CRPV-124	95344-95207			45			hypothetical protein, unique to CRPV, containing a transmembrane helix

CRPV Synteny	CRPV Genome Coordinates	CNPV Synteny	CNPV AA size	CRPV AA size	Best BLAST hits	CRPV AA Identity (%) compared to avipoxviruses	Notes
**CRPV-125**	**95804-95292**	**CNPV092**	**146**	**170**	**hypothetical protein [PEPV2]**	**98.4**	
**CRPV-126**	**96712-95906**	**CNPV093**	**267**	**268**	**SWPV2-088 virion protein**	**88.4**	
CRPV-127	96851-96759			30			hypothetical protein, unique to CRPV, containing a transmembrane helix
**CRPV-128**	**96814-97650**	**CNPV094**	**275**	**278**	**T10-like protein [FWPV]**	**96.4**	
CRPV-129	97800-97657	CNPV095	45	47	hypothetical protein [FWPV]	87.2	
CRPV-130	98037-97804	CNPV096	85	77	SWPV1-086 ubiquitin	100.0	
**CRPV-131**	**99106-98147**	**CNPV097**	**339**	**319**	**hypothetical protein [PEPV2]**	**92.9**	
CRPV-132	99365-99129	CNPV098	80	78	ChPV1-094 hypothetical protein	81.3	
CRPV-133	99958-99371	CNPV099	195	195	beta-NGF-like protein [FIPV]	95.2	
CRPV-134	100479-99982	CNPV100	169	165	SWPV2-095 putative interleukin binding protein	82.7	
CRPV-135	100617-100525			30			hypothetical protein, unique to CRPV
CRPV-136	101096-100782	CNPV102	105	104	SWPV2-097 conserved hypothetical protein	96.2	
**CRPV-137**	**101685-101113**	**CNPV103**	**190**	**190**	**CNPV103 N1R/p28-like protein**	**91.1**	
CRPV-138	101772-101945			57			hypothetical protein, unique to CRPV, containing a transmembrane helix
**CRPV-139**	**101896-102273**	**CNPV104**	**125**	**125**	**SWPV2-099 putative glutaredoxin 2, virion morphogenesis**	**99.2**	
**CRPV-140**	**102923-102216**	**CNPV106**	**234**	**235**	**transcriptional elongation factor [FIPV]**	**96.2**	
**CRPV-141**	**102917-103225**	**CNPV105**	**102**	**102**	**CNPV105 hypothetical protein**	**96.3**	
CRPV-142	103356-103577	CNPV107	77	73	CNPV107 hypothetical protein	80.0	

CRPV Synteny	CRPV Genome Coordinates	CNPV Synteny	CNPV AA size	CRPV AA size	Best BLAST hits	CRPV AA Identity (%) compared to avipoxviruses	Notes
**CRPV-143**	**103847-105742**	**CNPV108**	**632**	**631**	**CNPV108 putative metalloprotease**	**98.3**	
**CRPV-144**	**107771-105726**	**CNPV109**	**681**	**681**	**CNPV109 RNA helicase NPH-II**	**96.8**	
**CRPV-145**	**107806-109074**	**CNPV110**	**422**	**422**	**SWPV2-105 virion core proteinase**	**98.3**	
**CRPV-146**	**109079-110254**	**CNPV111**	**391**	**391**	**DNA-binding protein [MPPV]**	**99.0**	
**CRPV-147**	**110255-110500**	**CNPV112**	**81**	**81**	**CNPV112 putative IMV membrane protein**	**97.5**	
**CRPV-148**	**110522-111067**	**CNPV113**	**179**	**181**	**CNPV113 thymidine kinase**	**96.7**	
**CRPV-149**	**111196-111444**	**CNPV114**	**82**	**82**	**CNPV114 HT motif protein**	**100.0**	
**CRPV-150**	**111514-112383**	**CNPV115**	**289**	**289**	**DNA-binding phosphoprotein [MPPV]**	**98.6**	
**CRPV-151**	**112384-112593**	**CNPV116**	**69**	**69**	**conserved hypothetical protein [MPPV]**	**88.4**	
**CRPV-152**	**112600-113532**	**CNPV117**	**310**	**310**	**CNPV117 putative DNA-binding virion core protein**	**100.0**	
CRPV-153	113547-113651			34	fpO3L [MPPV]	97.1	
**CRPV-154**	**113712-115673**	**CNPV118**	**653**	**653**	**CNPV118 hypothetical protein**	**97.3**	
**CRPV-155**	**115603-115998**	**CNPV119**	**131**	**131**	**virion core protein [MPPV]**	**98.5**	
**CRPV-156**	**116276-115995**	**CNPV120**	**93**	**93**	**CNPV120 sulfhydryl oxidase**	**94.6**	
**CRPV-157**	**116303-119269**	**CNPV121**	**988**	**988**	**CNPV121 DNA polymerase**	**99.4**	
**CRPV-158**	**120085-119261**	**CNPV122**	**274**	**274**	**CNPV122 hypothetical protein**	**98.9**	
**CRPV-159**	**121802-120087**	**CNPV123**	**571**	**571**	**CNPV123 hypothetical protein**	**99.7**	
CRPV-160	127635-121864	CNPV124	1918	1923	variola B22R-like protein [PEPV2]	99.8	
CRPV-161	132948-127705	CNPV125	1767	1747	variola B22R-like protein [MPPV]	95.0	
CRPV-162	139065-133228	CNPV126	1951	1945	CNPV126 variola B22R-like protein	93.7	
**CRPV-163**	**139155-139703**	**CNPV127**	**182**	**182**	**CNPV127 RNA polymerase subunit RPO30**	**98.4**	
**CRPV-164**	**139735-141900**	**CNPV128**	**721**	**721**	**conserved hypothetical protein [MPPV]**	**96.4**	
**CRPV-165**	**141893-143311**	**CNPV129**	**472**	**472**	**poly(A) polymerase large subunit PAPL [MPPV]**	**98.7**	

CRPV Synteny	CRPV Genome Coordinates	CNPV Synteny	CNPV AA size	CRPV AA size	Best BLAST hits	CRPV AA Identity (%) compared to avipoxviruses	Notes
**CRPV-166**	**143661-143305**	**CNPV130**	**119**	**118**	**DNA binding virion core protein [FIPV]**	**98.3**	
**CRPV-167**	**143739-144356**	**CNPV131**	**207**	**205**	**SWPV2-126 conserved hypothetical protein**	**95.6**	
**CRPV-168**	**144450-144896**	**CNPV132**	**148**	**148**	**conserved hypothetical protein [MPPV]**	**98.0**	
CRPV-169	145131-145430	CNPV133	99	99	CNPV133 hypothetical protein	93.9	
CRPV-170	150902-145491	CNPV134	1801	1803	SWPV2-129 variola B22R-like protein	97.3	
**CRPV-171**	**151052-152188**	**CNPV135**	**378**	**378**	**SWPV2-130 putative palmitylated EEV envelope lipase**	**99.7**	
**CRPV-172**	**152266-154143**	**CNPV136**	**625**	**625**	**SWPV2-131 putative EEV maturation protein**	**96.8**	
**CRPV-173**	**154186-155565**	**CNPV137**	**465**	**459**	**SWPV2-132 conserved hypothetical protein**	**93.1**	
**CRPV-174**	**155656-156990**	**CNPV138**	**444**	**444**	**CNPV138 Ser/Thr kinase**	**97.8**	
**CRPV-175**	**156965-157606**	**CNPV139**	**213**	**213**	**conserved hypothetical protein [MPPV]**	**96.2**	
**CRPV-176**	**157687-157887**	**CNPV140**	**66**	**66**	**CNPV140 hypothetical protein**	**97.0**	
CRPV-177	157944-158063			39			hypothetical protein, unique to CRPV
CRPV-178	158213-158767	CNPV141	184	184	CNPV141 HAL3-like domain protein	93.5	
CRPV-179	159063-160016	CNPV142	321	317	KilA N domain protein [FIPV]	82.6	
CRPV-180	160126-160031			31			hypothetical protein, unique to CRPV
CRPV-181	160098-160595			165	ankyrin repeat containing protein [FIPV]	65.1	
CRPV-182	160721-160560			53	ankyrin repeat protein [MPPV]	72.9	
CRPV-183	160711-161721	CNPV143	671	336	ankyrin repeat containing protein [FIPV]	76.0	
CRPV-184	161945-162244			99	CNPV144 ankyrin repeat protein	85.9	
CRPV-185	162213-162404			63	ankyrin repeat protein [MPPV]	76.3	
CRPV-186	162736-162617			39	CNPV144 ankyrin repeat protein	81.6	
CRPV-187	163020-163139			39	ankyrin repeat containing protein [FIPV]	89.5	

CRPV Synteny	CRPV Genome Coordinates	CNPV Synteny	CNPV AA size	CRPV AA size	Best BLAST hits	CRPV AA Identity (%) compared to avipoxviruses	Notes
CRPV-188	163358-163552			64	ankyrin repeat protein [MPPV]	82.6	
**CRPV-189**	**163695-165023**	**CNPV145**	**440**	**442**	**CNPV145 Hypothetical protein**	**96.9**	
**CRPV-190**	**165031-165219**	**CNPV146**	**62**	**62**	**DNA-dependent RNA polymerase subunit [FIPV]**	**98.4**	
**CRPV-191**	**165212-165778**	**CNPV147**	**188**	**188**	**putative virion core protein [FIPV]**	**98.6**	
**CRPV-192**	**166789-165743**	**CNPV148**	**348**	**348**	**CNPV147 conserved hypothetical protein**	**99.5**	
**CRPV-193**	**167864-166950**	**CNPV149**	**306**	**304**	**putative thioredoxin binding protein [FIPV]**	**93.1**	
CRPV-194	168204-168323			39	CNPV150 ankyrin repeat protein	87.1	
CRPV-195	168354-168557	CNPV150	351	67	CNPV150 ankyrin repeat protein	65.2	
CRPV-196	168651-168538			37			hypothetical protein, unique to CRPV, containing a transmembrane helix
CRPV-197	170429-169191	CNPV151	412	412	CNPV151 ankyrin repeat protein	90.3	
CRPV-198	170823-170653	CNPV152	187	56	SWPV2-146 hypothetical protein	98.2	
CRPV-199	171018-171110			30			hypothetical protein, unique to CRPV
CRPV-200	171703-171215	CNPV153	312	162	SWPV2-147 Rep-like protein	83.1	
CRPV-201	171984-172088			34			hypothetical protein, unique to CRPV
CRPV-202	172412-172230			60	variola B22R-like protein [PEPV2]	94.8	
CRPV-203	172547-172443			34	variola B22R-like protein [PEPV2]	96.9	
CRPV-204	173492-172596	CNPV154	1928	298	CNPV154 variola B22R-like protein	89.0	
CRPV-205	174063-173461			200	variola B22R-like protein [PEPV2]	84.5	
CRPV-206	175520-174471	CNPV158	349	349	CNPV158 TGF-beta-like protein	95.1	
CRPV-207	175519-176037	CNPV157	172	172	TGF-beta-like protein	92.4	

CRPV Synteny	CRPV Genome Coordinates	CNPV Synteny	CNPV AA size	CRPV AA size	Best BLAST hits	CRPV AA Identity (%) compared to avipoxviruses	Notes
CRPV-208	176164-176072			30			hypothetical protein, unique to CRPV
CRPV-209	176163-176975	CNPV159	337	270	KilA N domain protein [FIPV]	91.7	
CRPV-210	177328-177218			36	N1R/p28-like protein [ChePV1]	96.3	
**CRPV-211**	**177347-178483**	**CNPV160**	**396**	**378**	**CNPV160 N1R/p28-like protein**	**94.7**	
CRPV-212	179653-178583	CNPV161	358	356	CNPV161 TGF-beta-like protein	98.3	
CRPV-213	179703-180152	CNPV162	149	149	SWPV2-153 TGF-beta-like protein	98.0	
CRPV-214	180496-181128			210	CNPV163 hypothetical protein	86.7	
**CRPV-215**	**181419-182462**	**CNPV165**	**346**	**347**	**N1R/p28-like protein [ALPV]**	**97.4**	
CRPV-216	182695-183741	CNPV166	345	348	CNPV166 Ig-like domain protein	92.4	
CRPV-217	184014-184553	CNPV167	171	179	immunoglobulin-like domain protein [FIPV]	73.7	
CRPV-218	185299-184598			233	CNPV199 deoxycytidine kinase-like protein	42.1	
CRPV-219	185412-185792			126	n1r/p28-like protein [ALPV]	85.2	
CRPV-220	185871-186509	CNPV170	212	212	CNPV170 thymidylate kinase	97.2	
**CRPV-221**	**186563-187345**	**CNPV171**	**260**	**260**	**CNPV171 late transcription factor VLTF-1**	**99.6**	
**CRPV-222**	**187359-188366**	**CNPV172**	**335**	**335**	**SWPV2-160 putative myristylated protein**	**95.6**	
**CRPV-223**	**188367-189098**	**CNPV173**	**243**	**243**	**putative myristylated envelope protein [FIPV]**	**97.5**	
**CRPV-224**	**189158-189448**	**CNPV174**	**96**	**96**	**CNPV174 conserved hypothetical protein**	**97.9**	
**CRPV-225**	**190349-189438**	**CNPV175**	**303**	**303**	**CNPV175 conserved hypothetical protein**	**99.0**	
**CRPV-226**	**190375-191133**	**CNPV176**	**252**	**252**	**CNPV176 DNA-binding virion core protein**	**99.2**	
**CRPV-227**	**191134-191526**	**CNPV177**	**130**	**130**	**L5 protein family protein [FIPV]**	**96.9**	
**CRPV-228**	**191480-191926**	**CNPV178**	**148**	**148**	**putative IMV membrane protein [MPPV]**	**100.0**	
**CRPV-229**	**191960-192868**	**CNPV179**	**302**	**302**	**CNPV179 poly(A) polymerase small subunit PAPS**	**96.4**	
**CRPV-230**	**192865-193425**	**CNPV180**	**186**	**186**	**CNPV180 RNA polymerase subunit RPO22**	**95.7**	

CRPV Synteny	CRPV Genome Coordinates	CNPV Synteny	CNPV AA size	CRPV AA size	Best BLAST hits	CRPV AA Identity (%) compared to avipoxviruses	Notes
**CRPV-231**	**193828-193418**	**CNPV181**	**136**	**136**	**CNPV181 conserved hypothetical protein**	**95.9**	
**CRPV-232**	**193872-197738**	**CNPV182**	**1288**	**1288**	**CNPV182 RNA polymerase subunit RPO147**	**98.8**	
**CRPV-233**	**198241-197741**	**CNPV183**	**166**	**166**	**CNPV183 putative protein-tyrosine phosphatase, virus assembly**	**98.8**	
**CRPV-234**	**198257-198826**	**CNPV184**	**189**	**189**	**CNPV184 putative viral membrane protein**	**100.0**	
CRPV-235	199081-198935			48	CNPV185 ankyrin repeat protein	95.8	
CRPV-236	199397-199179	CNPV185	328	72	SWPV2-173 ankyrin repeat protein	82.9	
CRPV-237	199727-199840			37	ankyrin repeat protein [MPPV]	93.3	
**CRPV-238**	**200829-199837**	**CNPV186**	**330**	**330**	**CNPV186 putative IMV envelope protein**	**97.0**	
**CRPV-239**	**203320-200921**	**CNPV187**	**799**	**799**	**RNA polymerase associated protein RAP94 [MPPV]**	**99.1**	
**CRPV-240**	**203488-204000**	**CNPV188**	**170**	**170**	**CNPV188 late transcription factor VLTF-4**	**91.2**	
**CRPV-241**	**204001-204951**	**CNPV189**	**316**	**316**	**SWPV2-177 DNA topoisomerase**	**99.1**	
**CRPV-242**	**204956-205417**	**CNPV190**	**153**	**153**	**SWPV2-178 conserved hypothetical protein**	**98.0**	
**CRPV-243**	**205691-205380**	**CNPV191**	**103**	**103**	**CNPV191 conserved hypothetical protein**	**94.2**	
**CRPV-244**	**205699-208236**	**CNPV192**	**846**	**845**	**CNPV192 mRNA capping enzyme large subunit**	**97.9**	
CRPV-245	208315-208629	CNPV193	106	104	HT motif protein [MPPV]	94.2	
**CRPV-246**	**209048-208626**	**CNPV194**	**140**	**140**	**CNPV194 virion protein**	**97.9**	
CRPV-247	209103-209246	CNPV195	144	47	SWPV2-183 hypothetical protein	70.4	
CRPV-248	209282-209437	CNPV195	144	51	CNPV195 hypothetical protein	75.9	
CRPV-249	209525-210043	CNPV196	190	172	CNPV196 hypothetical protein	92.9	
CRPV-250	210103-210918	CNPV197	275	271	N1R/p28-like protein [MPPV]	84.4	
CRPV-251	211544-211419			41	hypothetical protein [FIPV]	82.9	
**CRPV-252**	**211666-212343**	**CNPV199**	**225**	**225**	**CNPV199 deoxycytidine kinase-like protein**	**97.8**	

CRPV Synteny	CRPV Genome Coordinates	CNPV Synteny	CNPV AA size	CRPV AA size	Best BLAST hits	CRPV AA Identity (%) compared to avipoxviruses	Notes
CRPV-253	212749-212624			41	rep-like protein [PEPV2]	86.7	
CRPV-254	212887-213390	CNPV201	192	167	SWPV2-189 conserved hypothetical protein	90.4	
CRPV-255	213443-214273	CNPV202	276	276	CNPV202 N1R/p28-like protein	96.4	
CRPV-256	214346-215494	CNPV203	382	382	SWPV2-191 N1R/p28-like protein	92.7	
CRPV-257	215550-215717	CNPV204	61	55	CNPV204 conserved hypothetical protein	83.6	
CRPV-258	215898-216854	CNPV205	318	318	SWPV2-193 N1R/p28-like protein	95.6	
CRPV-259	216916-218334	CNPV206	472	472	CNPV206 putative photolyase	97.8	
CRPV-260	218430-218960	CNPV207	183	176	N1R/p28-like protein [MPPV]	91.2	
CRPV-261	219051-218953			32			hypothetical protein, unique to CRPV
CRPV-262	219065-219592	CNPV208	200	175	hypothetical protein [PEPV2]	92.1	
CRPV-263	219635-220567	CNPV209	310	310	CNPV209 N1R/p28-like protein	92.3	
CRPV-264	220616-221011	CNPV210	131	131	N1R/p28-like protein [MPPV]	97.7	
CRPV-265	221060-221224	CNPV211	54	54	SWPV2-199 conserved hypothetical protein	92.6	
CRPV-266	221278-221811	CNPV212	176	177	SWPV2-200 N1R/p28-like protein	91.0	
CRPV-267	222240-222515			91	deoxycytidine kinase-like protein [FIPV]	93.1	
CRPV-268	222620-223690	CNPV214	356	356	CNPV214 vaccinia C4L/C10L-like protein	92.4	
CRPV-269	223912-224526	CNPV215	204	204	SWPV2-203 CC chemokine-like protein	86.8	
CRPV-270	224622-225821	CNPV216	404	399	SWPV2-204 conserved hypothetical protein	92.0	
CRPV-271	226049-225834	CNPV217	330	71	CNPV217 N1R/p28-like protein	96.9	
CRPV-272	226156-226401	CNPV217	330	81	CNPV217 N1R/p28-like protein	88.9	
CRPV-273	226677-226450			75	SWPV2-205 N1R/p28-like protein	58.3	
CRPV-274	226864-228135	CNPV218	437	423	N1R/p28-like protein [PEPV2]	93.6	
CRPV-275	228398-228282			38			hypothetical protein, unique to CRPV

CRPV Synteny	CRPV Genome Coordinates	CNPV Synteny	CNPV AA size	CRPV AA size	Best BLAST hits	CRPV AA Identity (%) compared to avipoxviruses	Notes
CRPV-276	228693-228535			52			hypothetical protein, unique to CRPV, containing a transmembrane helix
CRPV-277	229090-230139	CNPV219	349	349	CNPV219 N1R/p28-like protein	97.7	
CRPV-278	230183-230392			69	KilA N domain protein [FIPV]	95.7	
CRPV-279	230734-230958			74	N1R/p28-like protein [ALPV]	84.9	
CRPV-280	231404-231288			38			hypothetical protein, unique to CRPV, containing a transmembrane helix
CRPV-281	232800-231505			431	ankyrin repeat protein [PEPV2]	75.2	
CRPV-282	233834-232794	CNPV223	847	346	ankyrin repeat protein [ChePV1]	92.0	
CRPV-283	234512-234417			31			hypothetical protein, unique to CRPV, containing a transmembrane helix
CRPV-284	234964-235209			81	N1R/p28-like protein [MLPV]	93.8	
CRPV-285	235206-235961			251	KilA N domain protein [FIPV]	81.1	
CRPV-286	237521-236217	CNPV229	434	434	ankyrin repeat protein [MPPV]	94.2	
CRPV-287	237512-237640			42			hypothetical protein, unique to CRPV
CRPV-288	237721-237918	CNPV230	65	65	hypothetical protein [MPPV]	95.4	
CRPV-289	237851-238330	CNPV231	158	159	CNPV231 MyD116-like domain protein	69.8	
CRPV-290	238364-238978	CNPV232	204	204	CC chemokine-like protein [ChePV1]	96.6	
CRPV-291	239126-240538	CNPV233	471	470	ankyrin repeat protein [MPPV]	94.9	
CRPV-292	240556-242082	CNPV234	508	508	CNPV234 ankyrin repeat protein	90.9	

CRPV Synteny	CRPV Genome Coordinates	CNPV Synteny	CNPV AA size	CRPV AA size	Best BLAST hits	CRPV AA Identity (%) compared to avipoxviruses	Notes
CRPV-293	242151-243443	CNPV235	432	430	conserved hypothetical protein [MPPV]	91.4	
CRPV-294	243488-244459	CNPV236	323	323	ribonucleotide reductase small subunit [MPPV]	98.5	
CRPV-295	244431-244544			37			hypothetical protein, unique to CRPV, containing a transmembrane helix
CRPV-296	244612-245910	CNPV237	441	432	ankyrin repeat containing protein [FIPV]	81.3	
**CRPV-297**	**246666-245989**	**CNPV238**	**225**	**225**	**CNPV238 late transcription factor VLTF-3**	**100.0**	
**CRPV-298**	**246881-246654**	**CNPV239**	**75**	**75**	**CNPV239 virus redox protein**	**96.0**	
**CRPV-299**	**248874-246895**	**CNPV240**	**659**	**659**	**CNPV240 virion core protein P4b**	**99.2**	
**CRPV-300**	**249608-248961**	**CNPV241**	**215**	**215**	**immunodominant virion protein [MPPV]**	**91.7**	
**CRPV-301**	**249647-250156**	**CNPV242**	**169**	**169**	**RNA polymerase subunit RPO19 [MPPV]**	**98.2**	
**CRPV-302**	**251272-250151**	**CNPV243**	**373**	**373**	**SWPV2-229 conserved hypothetical protein**	**98.1**	
**CRPV-303**	**253408-251279**	**CNPV244**	**709**	**709**	**early transcription factor large subunit VETFL [MPPV]**	**99.3**	
**CRPV-304**	**253472-254374**	**CNPV245**	**300**	**300**	**CNPV245 intermediate transcription factor VITF-3**	**99.0**	
**CRPV-305**	**254566-254336**	**CNPV246**	**75**	**76**	**putative IMV membrane protein [MPPV]**	**96.1**	
**CRPV-306**	**257248-254567**	**CNPV247**	**893**	**893**	**virion core protein P4a [MPPV]**	**98.3**	
**CRPV-307**	**257266-258090**	**CNPV248**	**279**	**274**	**CNPV248 conserved hypothetical protein**	**97.0**	
**CRPV-308**	**258593-258087**	**CNPV249**	**168**	**168**	**virion protein [MPPV]**	**97.0**	
CRPV-309	258608-258802	CNPV250	99	64	hypothetical protein [MLPV]	69.1	
**CRPV-310**	**259082-258873**	**CNPV251**	**69**	**69**	**putative IMV membrane protein [MPPV]**	**94.2**	
**CRPV-311**	**259408-259130**	**CNPV252**	**92**	**92**	**SWPV2-238 putative IMV membrane protein**	**89.1**	
CRPV-312	259586-259425	CNPV253	53	53	SWPV2-239 putative IMV membrane virulence factor	100.0	

CRPV Synteny	CRPV Genome Coordinates	CNPV Synteny	CNPV AA size	CRPV AA size	Best BLAST hits	CRPV AA Identity (%) compared to avipoxviruses	Notes
**CRPV-313**	**259892-259602**	**CNPV254**	**96**	**96**	**hypothetical protein CNPV254**	**97.9**	
**CRPV-314**	**260982-259876**	**CNPV255**	**368**	**368**	**CNPV255 predicted myristylated protein**	**97.0**	
**CRPV-315**	**261576-260998**	**CNPV256**	**192**	**192**	**CNPV256 putative phosphorylated IMV membrane protein**	**98.4**	
**CRPV-316**	**261594-262982**	**CNPV257**	**462**	**462**	**DNA helicase, transcriptional elongation [MPPV]**	**98.5**	
**CRPV-317**	**263210-262950**	**CNPV258**	**89**	**86**	**protein of unknown function (DUF678) [FIPV]**	**98.6**	
**CRPV-318**	**263556-263218**	**CNPV260**	**112**	**112**	**conserved hypothetical protein [MPPV]**	**96.8**	
**CRPV-319**	**263555-264856**	**CNPV259**	**434**	**433**	**CNPV259 DNA polymerase processivity factor**	**95.3**	
**CRPV-320**	**264856-265317**	**CNPV261**	**152**	**153**	**CNPV261 Holliday junction resolvase protein**	**94.8**	
**CRPV-321**	**265335-266486**	**CNPV262**	**383**	**383**	**intermediate transcription factor [FIPV]**	**95.8**	
**CRPV-322**	**266512-269985**	**CNPV263**	**1157**	**1157**	**CNPV263 RNA polymerase subunit RPO132**	**99.6**	
**CRPV-323**	**271761-269974**	**CNPV264**	**603**	**595**	**SWPV2-250 A type inclusion-like protein**	**95.5**	
**CRPV-324**	**273220-271796**	**CNPV265**	**475**	**474**	**A type inclusion-like/fusion protein [MPPV]**	**96.2**	
**CRPV-325**	**273643-273221**	**CNPV266**	**140**	**140**	**CNPV266 conserved hypothetical protein**	**95.0**	
**CRPV-326**	**274565-273648**	**CNPV267**	**305**	**305**	**DNA-directed RNA polymerase, 35 kD subunit [FIPV]**	**92.8**	
**CRPV-327**	**274764-274540**	**CNPV268**	**75**	**74**	**conserved hypothetical protein [MPPV]**	**97.4**	
CRPV-328	274889-275230	CNPV269	113	113	CNPV269 conserved hypothetical protein	93.8	
**CRPV-329**	**275239-275601**	**CNPV270**	**120**	**120**	**HGPV196 [Hawaiian goose poxvirus]**	**93.3**	
**CRPV-330**	**276444-275590**	**CNPV271**	**284**	**284**	**CNPV271 DNA packaging protein**	**96.8**	
CRPV-331	276589-276422			55	hypothetical protein [MPPV]	90.0	
**CRPV-332**	**276570-277115**	**CNPV272**	**181**	**181**	**CNPV272 C-type lectin-like EEV protein**	**96.1**	
**CRPV-333**	**277326-278150**	**CNPV273**	**274**	**274**	**CNPV273 conserved hypothetical protein**	**93.4**	
**CRPV-334**	**278211-279020**	**CNPV274**	**269**	**269**	**CNPV274 putative tyrosine protein kinase**	**92.2**	

CRPV Synteny	CRPV Genome Coordinates	CNPV Synteny	CNPV AA size	CRPV AA size	Best BLAST hits	CRPV AA Identity (%) compared to avipoxviruses	Notes
CRPV-335	279063-280079	CNPV275	338	338	SWPV2-261 putative serpin	96.5	
**CRPV-336**	**280884-280099**	**CNPV276**	**252**	**261**	**conserved hypothetical protein [MPPV]**	**89.7**	
CRPV-337	280994-281926	CNPV277	310	310	SWPV2-263 G protein-coupled receptor-like protein	97.1	
**CRPV-338**	**281937-282227**	**CNPV278**	**96**	**96**	**SWPV2-264 conserved hypothetical protein**	**97.9**	
CRPV-339	282295-282807	CNPV279	169	170	beta-NGF-like protein [MPPV]	90.4	
CRPV-340	283217-282825	CNPV280	130	130	HT motif protein [MPPV]	96.9	
**CRPV-341**	**283326-283955**	**CNPV281**	**214**	**209**	**SWPV2-267 conserved hypothetical protein**	**92.3**	
CRPV-342	284336-283974	CNPV282	120	120	HT motif protein [MPPV]	95.8	
CRPV-343	284502-284846	CNPV283	111	114	CNPV283 CC chemokine-like protein	96.3	
CRPV-344	284910-285491	CNPV284	195	193	putative interleukin binding protein [PEPV2]	86.0	
**CRPV-345**	**285601-285978**	**CNPV285**	**126**	**125**	**CNPV285 EGF-like protein**	**89.0**	
**CRPV-346**	**285980-286897**	**CNPV286**	**305**	**305**	**CNPV286 putative serine/threonine protein kinase**	**97.1**	
**CRPV-347**	**286940-287425**	**CNPV287**	**161**	**161**	**CNPV287 conserved hypothetical protein**	**96.9**	
CRPV-348	287461-287589			42	c-type lectin-like protein [PEPV2]	89.3	
CRPV-349	287699-287920	CNPV288	147	73	C type lectin domain protein [FIPV]	81.9	
**CRPV-350**	**287962-288381**	**CNPV289**	**139**	**139**	**putative interleukin binding protein [MPPV]**	**92.1**	
CRPV-351	288445-288672	CNPV290	75	75	CNPV290 conserved hypothetical protein	100.0	
CRPV-352	288875-290659	CNPV291	594	594	CNPV291 ankyrin repeat protein	94.8	
CRPV-353	290682-290906	CNPV292	74	74	CNPV292 hypothetical protein	96.0	
CRPV-354	290950-291804	CNPV293	284	284	SWPV2-279 ankyrin repeat protein	98.9	
CRPV-355	291859-293151	CNPV294	430	430	SWPV2-280 ankyrin repeat protein	98.6	
CRPV-356	293343-294533	CNPV295	396	396	CNPV295 ankyrin repeat protein	98.7	
**CRPV-357**	**294536-295912**	**CNPV296**	**458**	**458**	**CNPV296 ankyrin repeat protein**	**98.3**	

**CRPV Synteny**	**CRPV Genome Coordinates**	**CNPV Synteny**	**CNPV AA size**	**CRPV AA size**	**Best BLAST hits**	**CRPV AA Identity (%) compared to avipoxviruses**	**Notes**
CRPV-358	296016-296699	CNPV297	737	227	SWPV2-283 ankyrin repeat protein	95.6	
CRPV-359	296929-297381	CNPV297	737	150	CNPV297 ankyrin repeat protein	61.8	
CRPV-360	297519-299228	CNPV298	571	569	CNPV298 ankyrin repeat protein	96.2	
CRPV-361	299232-299951	CNPV299	300	239	CNPV299 putative serine/threonine protein kinase	84.9	
CRPV-362	300180-300073			35			hypothetical protein, unique to CRPV
CRPV-363	300202-300936	CNPV300	244	244	ankyrin repeat protein [MLPV]	95.1	
CRPV-364	301147-301242			31			hypothetical protein, unique to CRPV
CRPV-365	301433-301582			49	ankyrin repeat protein [MPPV]	81.1	
CRPV-366	301748-301596			50			hypothetical protein, unique to CRPV, containing a transmembrane helix
CRPV-367	301903-301730			57	ankyrin repeat containing protein [FIPV]	72.4	
CRPV-368	302219-302371			50			hypothetical protein, unique to CRPV
CRPV-369	302477-302298	CNPV301	527	59	SWPV2-287 ankyrin repeat protein	76.3	
CRPV-370	302639-302749			36	ankyrin repeat protein [MPPV]	90.6	
CRPV-371	303175-303011	CNPV302	193	54	hypothetical protein [FIPV]	79.6	
CRPV-372	303387-304889	CNPV303	500	500	ankyrin repeat protein [MPPV]	98.0	
CRPV-373	304898-305038			46			hypothetical protein, unique to CRPV
**CRPV-374**	**305114-306514**	**CNPV304**	**466**	**466**	**ankyrin repeat containing protein [FIPV]**	**90.6**	
CRPV-375	306584-307369	CNPV305	262	261	N1R/p28-like protein [MPPV]	97.3	
CRPV-376	307430-307648	CNPV306	72	72	CNPV306 hypothetical protein	93.1	

CRPV Synteny	CRPV Genome Coordinates	CNPV Synteny	CNPV AA size	CRPV AA size	Best BLAST hits	CRPV AA Identity (%) compared to avipoxviruses	Notes
CRPV-377	308119-307652	CNPV307	154	155	C-type lectin-like protein	90.3	
CRPV-378	308268-309350	CNPV308	357	360	SWPV2-294 ankyrin repeat protein	92.2	
CRPV-379	309495-310085	CNPV309	196	196	ankyrin repeat protein [MPPV]	96.9	
CRPV-380	310185-311834	CNPV310	537	549	ankyrin repeat protein [MPPV]	89.2	
CRPV-381	311859-312236	CNPV311	124	125	EFc gene family protein [FIPV]	90.4	
CRPV-382	312248-312748	CNPV312	166	166	conserved hypothetical protein [MPPV]	94.0	
CRPV-383	312817-313473	CNPV313	218	218	Ig-like domain protein [MPPV]	90.4	
CRPV-384	313635-315389	CNPV314	584	584	CNPV314 ankyrin repeat protein	96.4	
CRPV-385	315493-316458	CNPV315	315	321	G protein-coupled receptor-like protein [FIPV]	90.1	
CRPV-386	316508-318142	CNPV316	544	544	ankyrin repeat protein [PEPV2]	88.6	
CRPV-387	318607-319029			140	ankyrin repeat protein [MLPV]	54.1	
CRPV-388	319214-319399	CNPV319	739	61	SWPV2-305 ankyrin repeat protein	81.3	
CRPV-389	319347-319547			66	ankyrin repeat protein [PEPV2]	89.7	
CRPV-390	320107-320247	CNPV319	739	46	CNPV319 ankyrin repeat protein	93.5	
CRPV-391	320244-320354			36	ankyrin repeat containing protein [FIPV]	96.7	
CRPV-392	320448-320308	CNPV319	739	46	CNPV319 ankyrin repeat protein	85.1	
CRPV-393	320639-322051	CNPV320	469	470	SWPV2-306 Ig-like domain protein	90.9	
CRPV-394	322221-322583	CNPV321	124	120	EFc-like protein [MPPV]	83.6	
CRPV-395	322875-322988			37			hypothetical protein, unique to CRPV
CRPV-396	323255-323139			38			hypothetical protein, unique to CRPV
CRPV-397	323680-324018			112	ankyrin repeat protein [MLPV]	72.0	
CRPV-398	324146-324009			45	Ig-like domain protein [MLPV]	87.2	
CRPV-399	325442-324861	CNPV323	182	193	SWPV2-309 conserved hypothetical protein	84.5	

**CRPV Synteny**	**CRPV Genome Coordinates**	**CNPV Synteny**	**CNPV AA size**	**CRPV AA size**	**Best BLAST hits**	**CRPV AA Identity (%) compared to avipoxviruses**	**Notes**
CRPV-400	326070-325402	CNPV324	222	222	conserved hypothetical protein [MPPV]	97.3	
CRPV-401	326313-326981	CNPV325	514	222	CNPVCNPV325 ankyrin repeat protein	71.8	
CRPV-402	327008-327745	CNPV325	514	245	CNPVCNPV325 ankyrin repeat protein	55.1	
CRPV-403	328138-327662	CNPV327	171	158	hypothetical protein CNPV327	60.6	

Note: CRPV, crowpox virus; ALPV, albatrosspox virus; MLPV, mudlarkpox virus; MPPV, magpiepox virus; FGPV, flamingopox virus; PEPV, penguinpox virus; FeP2, pigeonpox virus; CNPV, canarypox virus; FIPV, finch poxvirus; FWPV, fowlpox virus.

Bold: The 89 core genes conserved in all ChPVs which are involved in essential functions such as replication, transcription and virion assembly.

Bold and blue: An additional 47 ORFs found to be uniquely conserved in the selected fully sequenced avian poxvirus genomes.

Supplementary Table S2. Number of ORFs in each of the 14 multigene families identified in the fully sequenced avian poxvirus genomes

Gene family	CRPV	ALPV2	ALPV	FWPV	MPPV2	MPPV	PEPV2	CNPV	SWPV2	MLPV	SWPV1	FP9	PEPV	FeP2	TKPV	FGPV
Ankyrin Repeat	73	33	48	31	78	62	49	51	46	47	50	22	33	26	16	45
B22R	8	6	6	6	9	7	6	6	7	7	6	5	5	4	1	4
N1R/p28	23	12	28	10	20	24	24	26	20	25	20	8	11	11	3	13
C4L/C10L	5	3	3	3	4	2	3	3	3	3	2	3	2	2	2	2
CC chemokine	4	4	5	4	7	4	5	5	5	5	6	4	1	4	2	6
C-type lectin	7	8	14	9	11	10	11	11	11	13	13	6	7	4	2	4
G protein-coupled receptor	4	3	4	3	4	4	4	4	4	4	4	2	3	2	2	3
HT motif	5	6	5	6	5	5	5	5	4	5	4	6	5	4	1	7
Ig-like domain	9	6	9	5	13	10	9	9	8	8	9	4	6	4	3	9
Serpin	5	6	5	5	5	5	5	5	5	5	5	5	4	4	3	5
EFc	2	3	2	3	3	2	2	2	2	1	2	2	1	1	1	1
TGF-β	6	1	5	1	5	4	5	5	4	6	3	1	1	1	1	1
β-NGF	2	2	2	2	2	2	2	2	2	2	2	2	0	0	2	3
IL-18 BP	3	2	3	1	3	3	3	3	3	3	3	1	1	0	2	0
**TOTAL**	**156**	**95**	**139**	**89**	**169**	**144**	**133**	**137**	**124**	**134**	**129**	**71**	**80**	**67**	**41**	**103**
